# AIEC-dependent pathogenic Th17 cell transdifferentiation in Crohn’s disease is suppressed by *rfaP* and *ybaT* deletion

**DOI:** 10.1080/19490976.2024.2380064

**Published:** 2024-07-29

**Authors:** G. Leccese, M. Chiara, I. Dusetti, D. Noviello, E. Billard, A. Bibi, G. Conte, C. Consolandi, M. Vecchi, MP Conte, N. Barnich, F. Caprioli, F. Facciotti, M. Paroni

**Affiliations:** a Department of Biosciences, Università degli Studi di Milano, Milan, Italy; b Gastroenterology and Endoscopy Unit, Fondazione IRCCS Ca’ Granda Ospedale Maggiore Policlinico, Milan, Italy; c Department of Pathophysiology and Transplantation, Università degli Studi di Milano, Milan, Italy; d M2iSH, UMR 1071 Inserm, INRAe USC 1382, CRNH, University of Clermont Auvergne, Clermont-Ferrand, France; e Institute of Biomedical Technologies, National Research Council (ITB-CNR), Segrate, Milan, Italy; f Department of Public Health and Infectious Diseases, ‘Sapienza’ University of Rome, Rome, Italy; g Department of Biotechnology and Bioscience, University of Milano-Bicocca, Milan, Italy

**Keywords:** pathogenic Th17 cells, Crohn’s disease, AIEC, IL-23

## Abstract

Mucosal enrichment of the Adherent-Invasive *E. coli* (AIEC) pathotype and the expansion of pathogenic IFNγ-producing Th17 (pTh17) cells have been linked to Crohn’s Disease (CD) pathogenesis. However, the molecular pathways underlying the AIEC-dependent pTh17 cell transdifferentiation in CD patients remain elusive. To this aim, we created and functionally screened a transposon AIEC mutant library of 10.058 mutants to identify the virulence determinants directly implicated in triggering IL-23 production and pTh17 cell generation. pTh17 cell transdifferentiation was assessed in functional assays by co-culturing AIEC-infected human dendritic cells (DCs) with autologous conventional Th17 (cTh17) cells isolated from blood of Healthy Donors (HD) or CD patients. AIEC triggered IL-23 hypersecretion and transdifferentiation of cTh17 into pTh17 cells selectively through the interaction with CD-derived DCs. Moreover, the chronic release of IL-23 by AIEC-colonized DCs required a continuous IL-23 neutralization to significantly reduce the AIEC-dependent pTh17 cell differentiation. The multi-step screenings of the AIEC mutant’s library revealed that deletion of *ybaT* or *rfaP* efficiently hinder the IL-23 hypersecretion and hampered the AIEC-dependent skewing of protective cTh17 into pathogenic IFNγ-producing pTh17 cells. Overall, our findings indicate that *ybaT* (inner membrane transport protein) and *rfaP* (LPS-core heptose kinase) represent novel and attractive candidate targets to prevent chronic intestinal inflammation in CD.

## Graphical abstract

**Figure:**
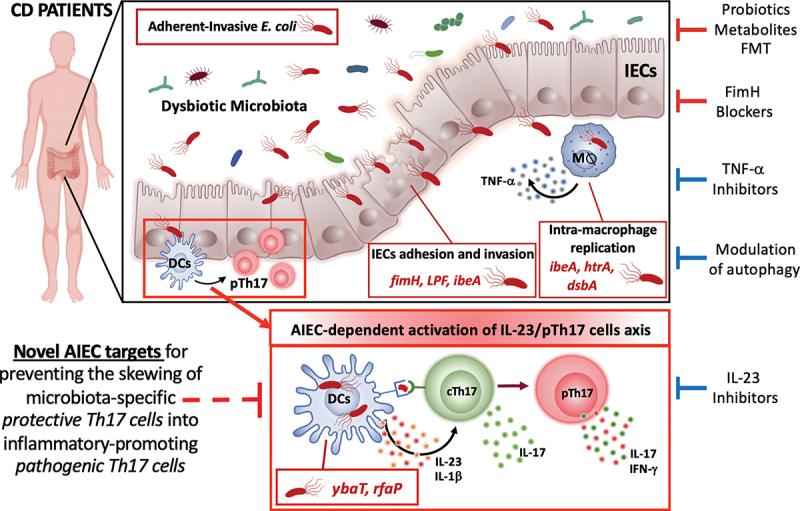

## 1. Introduction

Crohn’s disease (CD) is a chronic inflammatory and multifactorial non-communicable disease affecting the digestive tract, characterized by a dysregulated activation of the mucosal immune system in response to a dysbiotic gut microbiota.^1^ Current therapies in CD mostly target effector immune responses and are characterized by a non-optimal net remission rate,^2^ due to primary non-response or secondary loss of response.^3^ Microbiota-based therapies^4,5^ to restore normobiosis are still in an early stage of clinical investigation, and their development is currently limited by the knowledge gap regarding the activation of microbiota-specific immune cells.^6^

Intestinal inflammation in CD has been classically linked to an altered activation of tissue-resident memory (T_RM_) Th1 and Th17 cells. However, while the protective role of Th1 in response to intracellular pathogens has been clearly established, the role of T_RM_ Th17 cells in CD has long been debated. Clinical evidence and murine models of colitis reported both protective and pathogenic functions for intestinal Th17 cells. Colonization of germ-free mice with commensal bacteria results in the generation of intestinal-resident homeostatic conventional Th17 cells with a non-inflammatory cytokine profile and a protective function for the mucosal barrier ^7–9^. In contrast, intestinal infection of mice with bacterial pathogens^7,10^ or microbiota transplantation from CD patients^11^ promotes the differentiation of IFNγ-producing Th17 cells with a pro-inflammatory profile and pathogenic functions. High IL-23 levels have been demonstrated to be necessary to shift Th17 cells toward a pathogenic IFNγ-producing profile in several experimental models of autoimmune and inflammatory conditions.^12–14^

Distinct Th17 cell populations with opposite functions have also been described in human,^15,16^ with a clear pathogenic role in several autoimmune diseases including CD.^17,18^ Enrichment of IFNγ/IL-17 co-producing Th17 cells with a pathogenic profile has been reported in the intestinal mucosa of CD patients.^19–21^ Moreover, in contrast to the failure of anti-IL-17 therapies,^22^ anti-IL-23p19 biologicals showed better clinical response and remission rates in CD patients.^23^

We recently described the presence, induction, and pathogenic functions of a novel subset of T_RM_ IFNγ-producing Th17 cells (pTh17), which are selectively increased in the intestinal tissues of CD patients, induced by high amount of IL-23, and strongly reduced following anti-IL-23 treatment.^24^ Functionally, these pTh17 cells are specifically activated by Adherent-Invasive *E. coli* (AIEC) strains, thus sustaining the hypothesis that T cells overactivation in CD stems from a loss of tolerance to enteric microbiota.

Within the dysbiotic CD gut microbiota AIEC has been pointed out as one of the most concerning determinants in CD pathogenesis,^25^ being highly prevalent in the ileal mucosa of CD patients^26,27^ and colitogenic in several murine models.^28^ The virulence of AIEC strains known nowadays rely on their ability to adhere and invade intestinal epithelial cells (IECs),^29^ and extensively survive within macrophages secreting high levels of tumor necrosis factor alpha (TNFα).^30^ However, while the interplay between AIEC and intestinal epithelial cells or macrophages has been extensively characterized,^31–34^ the molecular mechanisms behind the AIEC-antigen dependent activation of pTh17 cells are completely unknown.

Here, we combined microbial genetics approaches with functional immunological assays to identify specific AIEC determinants triggering the hypersecretion of IL-23 and the exaggerated pTh17 cell activation in CD patients. We demonstrated that AIEC directly promotes the transdifferentiation of pathogenic IFNγ-producing pTh17 cells from the precursors non-pathogenic Th17 cells. By screening a library of 10.058 mutants, we identified a restricted set of AIEC genes involved in triggering IL-23 hyperproduction by CD-derived dendritic cells (DCs).

Finally, we demonstrated that inhibiting AIEC pathways associated with LPS core biosynthesis (*rfaP*), and the inner membrane transport protein (*ybaT*), hampered the IL-23 overly secretion by DCs and strongly hinder the AIEC-dependent generation of pTh17 cells. This effect is similar to that observed with a continuous IL-23 neutralization, thus providing for the first time a rationale for a functional targeting of the AIEC determinants triggering pathogenic pTh17 cells in CD patients.

## 2. Materials and methods

### 2.1. Patients

Clinical characteristics of CD patients and HD included in this study are listed in **Table S1**. The HD cohort comprises 47 HD with no personal or family history of allergies or immune-mediated diseases. The CD cohort includes 44 patients with a confirmed diagnosis of CD for at least 6 months according to the current ECCO guidelines either in clinical remission or with a mild clinical activity according to the Harvey-Bradshaw index (HBI) enrolled in the IBD outpatient clinic of the Foundation IRCCS Ca’ Granda, Ospedale Maggiore Policlinico, Milan, Italy, during scheduled visits according to clinical practice. Subjects taking oral corticosteroids in the preceding 4 weeks, immunomodulators in the preceding 3 months, biologics in the preceding 12 weeks, or antibiotics in the preceding 4 weeks were excluded. The studies involving human participants were carried out in compliance with the Declaration of Helsinki protocols and were reviewed and approved by the Ethics commission (Milano Area B, code 128_2018bis).

### 2.2. Bacterial strains and generation of transposon library of AIEC-LF82 mutants

The AIEC bacterial strains used in this project are listed in **Table S2**. The probiotic *E. coli* Nissle 1917 strain (EcN, Mutaflor; DMS 6601), belonging to the same phylogroup of LF82, was used as non-pathogenic control. Bacteria were grown in YESCA medium,^35^ unless otherwise indicated.

A Tn5 transposon library of 10.058 AIEC-LF82 kanamycin-resistant mutants was generated using EZ-Tn5™ <R6Kγori/KAN-2>Tnp Transposome™ Kit (Lucigen) following the manufacturer’s protocol. LF82 mutants were grown in YESCA supplemented with kanamycin (50 μg/ml). The mutated genes in selected LF82 mutants were identified by rescue cloning according to the manufacturer’s instructions and sequenced by Eurofins Sanger Sequencing Services. For the identification of the transposon insertion point, the sequencing results were aligned on the LF82 genome (sequence ID: CU651637) with BLAST program (NCBI).

LF82-*ΔfimH*, -*ΔrfaP*, and -*ΔybaT* isogenic deletion mutants were generated using the λ-Red recombination system^36,37^ with the primers listed in **Table S3**. For subsequent infection experiments, antibiotic resistance was removed transforming mutants with pCP20 plasmid,^36^ primers used to confirm target gene deletion are listed in **Table S3.**

### 2.3. Human primary cells isolation and cultures

Monocyte-derived dendritic cells (moDCs), monocyte-derived macrophages (MDMs) and Human intestinal epithelial HT29 cell line (IECs) were differentiated and cultured as previously described.^35^ Th1 and Th17 cell subsets were sort purified (FACSAria III, BD Biosciences) from peripheral blood mononuclear cells (PBMC) of HD and CD patients according to the expression of specific surface markers combination^24:^ Th1: CD4^+^IL-7 R^+^CD25^low^CCR6^–^CXCR3^+^; conventional cTh17 cells: CD4^+^IL-7 R^+^CD25^low^CCR6^+^CXCR3^–^CCR5^–^; pathogenic pTh17 cells: CD4^+^IL-7 R^+^CD25^low^CCR6^+^CXCR3^–^CCR5^+^ (gating strategy reported in Figure S1b).

### 2.4. Infection assays and antigen-specificity assays

HD- and CD-derived MDMs or moDCs were infected with 10^7^ CFU/ml of bacterial strain (Multiplicity of infection-MOI 1:10). Bacterial phagocytosis and intracellular persistence were evaluated as previously described.^35^ When stated 40 μg/ml of neutralizing human anti-TLR4 (clone W7C11; invivogen) was added to moDCs 1 h before bacterial infection. HT29 cells were infected with LF82 or LF82 mutants at the final concentration of 1.4 × 10^6^ CFU/well. At 24 h post infection, supernatants of infected cells were collected and stored at −20°C for cytokine quantification by ELISA assay.

AIEC-dependent T-cell transdifferentiation into IFNγ-producing Th17 cells (pTh17) was analyzed by co-culturing 5 × 10^4^ autologous cTh17 or Th1 cells with LF82- or LF82 mutants-infected moDCs or MDMs at a 5:1 ratio for 10 d in RPMI medium supplemented with 2 μg/ml gentamycin and 20 U/ml of recombinant human IL-2 (rhIL-2). For proliferation assay, cTh17 cells were first labeled with 5 μM Carboxyfluorescein succinimidyl ester (CFSE; Biolegend) and proliferating cells (CFSE dye dilution) were analyzed by flow cytometry. Background proliferation induced by autologous unstimulated moDCs was subtracted to calculate antigen-specific proliferation. For AIEC-dependent pTh17 cell transdifferentiation, co-cultures of T cells with infected moDCs or MDMs were analyzed for intracellular cytokine expression after a further polyclonal stimulation with PMA/Ionomycin as previously described.^24^ After fixation and permeabilization, T cells were stained with anti-CD40L, anti-IL-17, and anti-IFNγ (Biolegend). Background activation of T cells stimulated with autologous uninfected moDCs or MDMs was subtracted to calculate antigen-dependent pTh17 cell transdifferentiation. In some experiments neutralizing anti-IL-23 (clone HNU2319; Invitrogen) and/or anti-IL-1β antibodies (clone CRM56; Invitrogen) were added together with cTh17 cells at *T* = 0 or every 48 h during the assay. Mouse IgG1k (clone P3.6.2.8.1; Invitrogen) has been used as isotype control. Acquisition was performed with FACSCanto II cytometer and data were analyzed using FlowJo software (BD Biosciences).

### 2.5. Functional screenings of AIEC-LF82 mutant library

For the first screening, the parental LF82 strain and LF82 mutants were individually grown in YESCA medium in 96 well plates O/N at 37°C in an orbital shaking. Bacterial concentration of LF82 and of each single mutant were evaluated in a microplate reader (SAFAS MP96) and, thereafter, the same bacterial concentration calculated on the correspondence of OD_600_–CFU/ml for the parental LF82 strain was used to infect human cells with each single LF82 mutant strain resuspended in complete RPMI without antibiotics.

The amount of IL-23 (Invitrogen) and IL-1β (BioLegend) released by moDCs, as well as CCL20 (MIP-3α; R&D Systems) released by HT29 cells, after 24 h of infection with each single mutant (M) was quantified by ELISA assays. Cytokine levels were normalized on the growth of each mutant strain (OD_600_ of the O/N bacterial cultures) and compared with the LF82 reference strain using the following formula:

Norm C/GR = log_2_ (C_M_/GR_M_) – log2(C_LF82_/GR_LF82_)

where C is the cytokine concentration (OD_450_) released in response to each single mutant (C_M_) or to the parental LF82 strain (C_LF82_); GR is the corresponding growth rate (OD_600_) of each mutant (GR_M_) or of the LF82 (GR_LF82_).

The top 1% mutants associated with a reduced IL-23 production were selected for subsequent screenings by statistical analysis of the values distribution. Mutant strains in essential genes or with a defective growth rate as compared to the parental LF82 strain were discarded.

From the second screening onward, to obtain a strict specific MOI of 1:10, selected mutants were individually normalized on the specific correspondence of OD_600_–CFU/ml.

### 2.6. *Phylogenetic distribution of selected genes across* E. coli *pathotypes and phylogroups*

*E. coli* pathotypes and phylogroups were assigned according to the definitions of Clark et al.^38^ Complete genome sequences, and corresponding genome annotations were retrieved from GenBank.^39^ Gene presence/absence profiles were determined by protein sequence similarity searches based on blastp with default parameters^40^; sequences derived from the reference assembly of the LF82 strain were used as a query. A gene was considered to be present in a reference genome assembly if/when the following criteria were fulfilled: level of identity with the query sequence >80%; the alignment covered at least 75% of the size of the query sequence.

### 2.7. Phenotypic characterization of AIEC-LF82 mutant strains

For assessing differential bacterial growth in a medium that mimic the phagolysosome vacuole content, O/N cultures of LF82 and LF82 mutants were diluted to an OD_600_ = 0.02 in the Acid and Nutrient-poor medium (ANp)^33^ and bacterial growth curves were evaluated for 16 h in a microplate reader (SAFAS MP96) which ensures shaking conditions and constant temperature. Biofilm formation (crystal violet assay),^41^ flagellar motility,^42^ Type 1 fimbriae expression (FimH, yeast aggregation assays)^42^ and curli (congo red assay)^43^ were evaluated as previously described.

### 2.8. Statistical analysis

Statistical significance was assessed through paired or unpaired t-test for comparison of two groups (applying Mann-Whitney’s or Welch’s correction for non-normally distributed variables) or by one-way ANOVA for comparison of more than two groups (with Dunn’s multiple comparisons for non-normally distributed variables). All experiments were performed at least 3 times. Statistics were performed with Prism software (version 9; GraphPad Software) and indicated in the respective figure legends.

## 3. Results

### 3.1. Interplay between AIEC and CD-derived dendritic cells promoted the overly secretion of IL-23 and transdifferentiation of pTh17 cells

**Figure 1.**
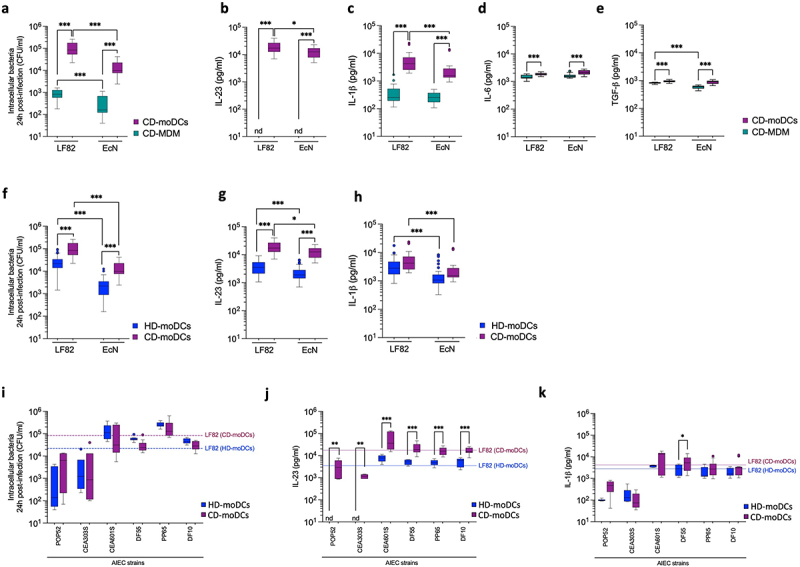
AIEC intracellular persistence within dendritic cells promotes dysregulated IL-23 hypersecretion selectively in CD patients.

We functionally evaluated the inflammatory response of AIEC-infected MDMs and moDCs isolated from the peripheral blood of CD patients, focusing on IL-23 and IL-1β secretion for their important polarizing role in pTh17 cell differentiation.^14,44–46^ The probiotic EcN strain, belonging to the same phylogroup of LF82, was used as nonpathogenic control (Figure 1).

(a) Intracellular bacterial load of AIEC-LF82 and the probiotic Nissle 1917 (EcN) strains after 24 h of infection within monocyte-derived macrophages (MDMs, *n* = 6) or monocyte-derived dendritic cells (moDCs, *n* = 10) isolated from CD patients.

(b) IL-23, (c) IL-1β, d) IL-6, and (e) TGF-β secretion by CD-derived MDMs (*n* = 6) or CD-derived moDCs (*n* = 10) after 24 h of infection with LF82 or EcN strains was measured by ELISA.

(f) Intracellular bacterial load of LF82 or EcN strains within moDCs isolated from CD patients (*n* = 10) or healthy donors (HD, *n* = 15), at 24 h post-infection.

(g) IL-23 and (h) IL-1β secretion by CD-derived (*n* = 10) or HD-derived (*n* = 15) moDCs at 24 h post-infection with LF82 or EcN strains was measured by ELISA.

(a-h) Statistical significance was calculated using Mann-Whitney test and reported as *p < 0.05, ** *p* < 0.01, *** *p* < 0.001.

(i) Intracellular bacterial load of LF82 and other indicated clinical AIEC strains within moDCs isolated from CD patients (*n* = 3–4) or HD (*n* = 3–4), at 24 h post-infection. Horizontal lines represent the median of the intracellular bacterial load of LF82 within CD-moDCs (purple line) or within HD-moDCs (blue line); respectively.

(j) IL-23 and (k) IL-1β production by CD-derived (*n* = 3–4) or HD-derived moDCs (*n* = 3–4) at 24 h post-infection with LF82 or other indicated clinical AIEC strains. Horizontal lines represent the median of IL-23 or IL-1β released by CD-moDCs (purple line) or by HD-moDCs (blue line), respectively, in response to LF82.

(i-k) Statistical significance was calculated by comparing CD- and HD-derived moDCs infected with the indicated AIEC strains using Mann–Whitney test, and reported as: *p < 0.05, ** *p* < 0.01, *** *p* < 0.001.

LF82 persisted significantly more than EcN within both MDMs and moDCs at 24 h post infection, with a significantly higher intracellular bacterial load within moDCs (Figure 1a). At this time point, LF82 triggered hypersecretion of IL-23 exclusively by moDCs (Figure 1b) and promoted a significantly higher release of IL-1β by moDCs, but lower than IL-23, compared to MDMs (Figure 1c). Interestingly also EcN triggered the secretion of pro-inflammatory IL-23 and IL-1β. Instead, no difference in the secretion of TGF-β and IL-6, which have a more prominent role in the differentiation of naive CD4^+^ T cells to cTh17 cells,^47^ was observed in response to LF82 or EcN by moDCs or MDM derived from CD patients (Figures 1d,e).

LF82 and EcN strains persisted significantly more within CD-derived moDCs than in those derived from healthy donors (HD) (Figure 1f), like previous observations in CD-macrophages.^48^ A dysregulated inflammatory response selectively in CD patients was confirmed by the secretion of IL-23, but not of IL-1β, by CD- compared to HD-moDCs (Figure 1g,h) and slightly higher levels of IL-6 and TGF-β (Figure S1a) in response to LF82 and EcN. Similar results, were confirmed upon DCs exposure to other AIEC clinical strains isolated from CD patients (Figures 1i-k). Indeed, although AIEC clinical isolates differentially persisted within DCs as compared to the reference LF82 strain (Figure 1i), they all promoted similar IL-1β amount from HD- and CD-derived DCs (Figure 1k) but a significant IL-23 overproduction selectively by CD-moDCs (Figure 1j).

**Figure 2.**
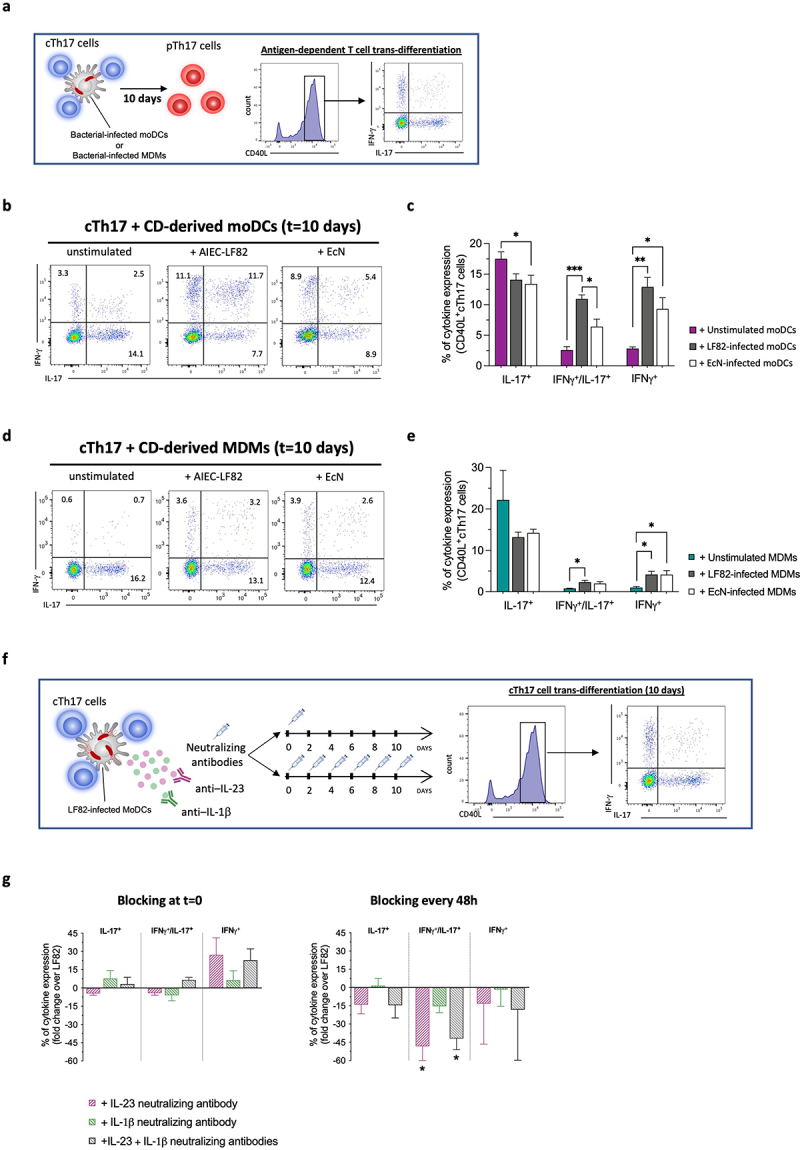
AIEC strongly promotes the transdifferentiation of conventional Th17 cells into pathogenic IFNγ-producing pTh17 cells selectively through the interaction with CD-derived DCs.

Next, we assessed whether AIEC was directly implicated in the transdifferentiation of human pathogenic IFNγ-producing pTh17 cells, employing the well-established antigen-dependent functional assay (Figure 2a). Exposure of human conventional Th17 cells (cTh17), but not of Th1 cells (Figure S1b), to LF82-infected CD-moDCs induced a sustained co-production of IFNγ/IL-17 (Figures 2b,c and S1d), resembling ex vivo activated pTh17 cells (Figure S1c). In contrast, EcN-infected moDCs induced a phenotype more similar to unstimulated T cells, despite the high IL-23 levels released by EcN-stimulated CD-moDCs (Figure 2b and 1b). Moreover, precursors cTh17 cells shifted toward IFNγ-producing pTh17 cells exclusively upon exposure to LF82-infected moDCs derived from CD patients but not from HD (Figure S1e). Importantly, MDMs derived from CD patients did not secrete IL-23 in response to *E. coli* strains tested (Figure 1b) and failed to induce transdifferentiation of cTh17 cells into pTh17 cell*s* (Figures 2d,e), thus indicating a more prominent role of dendritic cells in the AIEC-dependent pTh17 cell generation.

(a) Experimental strategy to analyze the antigen-dependent transdifferentiation of conventional Th17 cells (cTh17) into pathogenic IFNγ-producing Th17 cells (pTh17 cells).

Peripheral cTh17 cells sort-purified from blood of CD patients were stimulated with autologous moDCs (b-c; *n* = 6) or MDMs (d-e; *n* = 4) alone (unstimulated), or infected with AIEC-LF82, or EcN strains. After 10 d of co-culture, expression of IFNγ, IL-17 and co-expression of IFNγ/IL-17 was measured by intracellular staining.

(b and d) One representative experiment showing the gating strategy to identify antigen-dependent pTh17 transdifferentiation is shown.

(c and e) Mean percentage of single expression of IFNγ^+^, IL-17^+^ and IFNγ^+^/IL-17^+^ co-expression on CD40L^+^cTh17 cells after 10 d of co-culture with indicated stimuli were reported. Statistical significance was calculated using one-way ANOVA and reported as *p < 0.05, ** *p* < 0.01, *** *p* < 0.001.

(f) Experimental strategy to analyze the effect of single or continuous IL-23 and/or IL-1β blocking on the AIEC-dependent transdifferentiation of cTh17 into pTh17 cells.

(g) Fold-change values of the percentages of single expression of IFNγ^+^, IL-17^+^ and IFNγ^+^/IL-17^+^ co-expression on peripheral CD40L^+^cTh17 cells after 10 d of co-culture with autologous CD-derived moDCs infected with LF82 in the presence of indicated neutralizing antibodies added at *t* = 0 (**left graph**) or every 48 h (**right graph**). Data are represented as mean percentage (fold-change over LF82) ± SEM of at least three independent experiments. Statistical significance was calculated using one-way ANOVA and reported as *p < 0.05, ** *p* < 0.01, *** *p* < 0.001.

To demonstrate the functional roles of IL-23 and IL-1β in AIEC-dependent pTh17 cell generation we added neutralizing antibodies against IL-23 and/or IL-1β at different time points during the in vitro pTh17 transdifferentiation assay (Figure 2f). Single blockade of IL-23 or IL-1β at day 0 only slightly reduced the IFNγ/IL-17 co-expression by cTh17 cells in response to LF82-stimulated DCs (Figure 2g-left graph). Conversely, a continuous IL-23 blocking by adding neutralizing antibodies every 48 h resulted in a sharp reduction of IFNγ/IL-17 co-expression by AIEC-exposed cTh17 cells (Figure 2g-right graph). No difference was observed between untreated cells and treated cells with isotype control every 48 h (data not shown).

Taken together, these results indicated that AIEC interacts differently with DCs derived from CD or HD triggering a sustained IL-23 hypersecretion and the transdifferentiation of pTh17 cells from precursors protective cTh17 cells exclusively in CD patients.

### 3.2. Functional screening of a LF82 mutant library identified AIEC determinants specifically involved in triggering IL-23 hypersecretion by DCs

**Figure 3.**
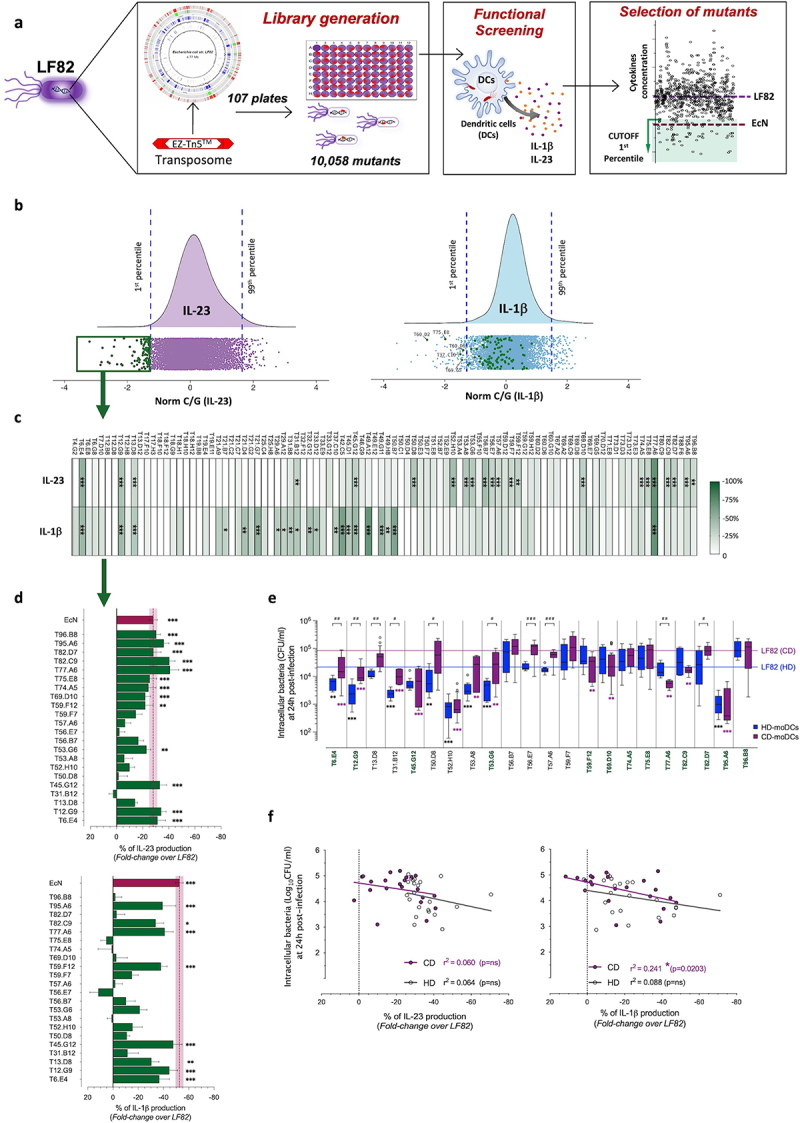
Immunological and functional screening of the AIEC-LF82 mutant library on HD-and CD-derived DCs.

We then aimed at identifying the AIEC determinants triggering the transdifferentiation of cTh17 cells into pTh17 cells. We generated and functionally screened a library of 10.058 LF82 mutants, carrying a single transposon insertion compared to the parental LF82 strain, using the EZ-Tn5™ <R6Kγori/KAN-2>Tnp Transposome system (Figure 3a). Having demonstrated the crucial polarizing role of IL-23 as downstream signal during antigen-dependent pTh17 cell activation, we performed a multi-step screening assay using the IL-23 levels released by AIEC-infected moDCs as readout. The multi-step screening was consequent of the technical impossibility to carry out the antigen-specificity assay with 10 thousand mutants directly on CD-derived immune cells (isolated from a limited amount of peripheral blood). Thus, in the first step, all mutants were individually tested on buffy-coat derived HD-moDCs and those displaying the highest reduction in IL-23 secretion (under the 1^st^ percentile, *p* < 0.01 as compared to the parental LF82 strain), were selected for the second step of screening (Figure 3b-left graph).

(a) Experimental design for generating and screening the AIEC-LF82 transposon insertion library and the subsequent selection of mutants as described in material and methods.

(b) First screening: Distribution of growth-normalized IL-23 (**left graph**) and IL-1β (**right graph**) production, log scaled (normC/G), measured in the supernatants of HD-derived DCs after 24 h of infection with each single LF82 mutant in comparison to the parental LF82 strain taken as zero. Dots represent individual measures for each mutant, and the inferred probability density function is represented at the top. Vertical dotted blue lines indicate the 1^st^ and 99^th^ percentile, respectively. Green dots outline the 96 selected LF82 mutants with a normC/G IL-23 value below the 1^st^ percentile (**left graph**), and their corresponding IL-1β values (**right graph**), calculated with the normalization formula reported in the material and methods. Probability density functions were inferred using the density() function from the R stats package. Graphical representation of the data by ggplot2 R library. R version 4.2.1 was used.

(c) Second screening: fold-change values, represented as heatmap, of the percentage of IL-23 and IL-1β (rows) of the 96 selected LF82 mutants (columns), in comparison to the parental LF82 strain taken as zero, tested on HD-moDCs (*n* = 3–5 independent experiments) after a strict normalization of each single mutant on the corresponding CFU/ml value. Statistical significance between LF82 and LF82 mutants was calculated using one-way ANOVA and reported as: *p < 0.05, ** *p* < 0.01, *** *p* < 0.001.

(d) Third screening: fold-change values of IL-23 (**upper graph**) and IL-1β (**lower graph**) of the 22 selected LF82 mutants after the second screening, in comparison to the parental LF82 strain taken as zero, tested on CD-moDCs (*n* = 5). Data are represented as mean percentage (fold-change over LF82) ± SEM of at least 3–5 independent experiments. Vertical dotted purple lines indicate the mean ± SEM of fold-change values for the probiotic EcN strain. Statistical significance was calculated using one-way ANOVA and reported as: *p < 0.05, ** *p* < 0.01, *** *p* < 0.001.

(e) Intracellular bacterial load (CFU/ml) of LF82 mutants within moDCs isolated from CD patients (*n* = 5; purple bars) or HD (blue bars) after 24 h of infection. Data are represented as mean ± SEM of at least 3–5 independent experiments. Horizontal lines indicate the mean of LF82 intracellular bacterial load within CD-derived (purple line) or HD-derived moDCs (blue line). LF82 mutants with an impaired ability to trigger IL-23 secretion by CD-moDCs are marked in green. Statistical significance between values of LF82 mutants and the parental LF82 strain was calculated using one-way ANOVA and reported as *p < 0.05, ** *p* < 0.01, *** *p* < 0.001 (in black for the comparison among HD-moDCs, in purple for the comparison among CD-moDCs). Statistical significance between values of the same LF82 mutant in DCs derived from HD vs CD patients was calculated using unpaired t-test and reported as: #p < 0.05, ## *p* < 0.01, ### *p* < 0.001.

(f) Correlation between intracellular persistence of LF82 mutants within HD- (white dots) or CD-moDCs (purple dots) at 24 h post-infection and fold changes in the percentage of IL-23 (**left graph**) and IL-1β (**right graph**) production of each LF82 mutant, as compared to the parental LF82 strain. Correlations were determined using Pearson correlation coefficients. R squares and *p* values for each correlation were reported in the graphs.

As comparative control, given the important role of CCL-20 in recruiting DCs and Th17 cells at the site of infection, we checked if AIEC mutants also affected CCL-20 released by intestinal epithelial HT29 cells (IECs).

Among 96 selected mutants inducing a significant IL-23 reduction (Figure 3b-left graph), 5 mutants (5.2%) also induced a significant reduction of IL-1β secretion by HD-moDCs (Figure 3b-right graph), while 10 distinct mutants (9.4%) also a reduced CCL20 secretion by IECs compared to the parental LF82 strain (Figure S2a) thus indicating that these three inflammatory pathways are stimulated by different AIEC determinants.

As second screening, to exclude the selection of false-positive mutants based on an underestimated infection rate, a more stringent normalization was applied for each single mutant, by evaluating the correspondence between OD_600_ and bacterial cell count (CFU/ml) and the probiotic EcN strain was used as reference threshold. Results revealed that, when tested at the effective MOI of 1:10, 22 out of the initial 96 LF82 mutants triggered the secretion of similar or even significantly lower levels of IL-23 by HD-moDCs as compared to EcN (Figure 3c and S2b). Again, among these 22 strains, 6 mutants also triggered a significantly lower IL-1β secretion by HD-moDCs, resembling the non-pathogenic inflammatory response following EcN infection, while 15 distinct mutants out of 96 induced only a lower secretion of IL-1β but not of IL-23 (Figure 3c and S2c).

Next, as third screening we tested these 22 selected LF82 mutants on CD-moDCs. Results showed that 13 out of 22 mutants stimulated a lower secretion of IL-23 (Figure 3d-upper graph), and 8 of them also lower levels of IL-1β by CD-moDCs, compared to the parental LF82 strain (Figure 3d-lower graph). To evaluate whether the reduced pro-inflammatory response was associated with a reduced bacterial survival within DCs, we analyzed the intracellular bacterial load of LF82 mutants within HD- and CD-derived moDCs at 24 h post infection, as well as the bacterial growth in an acid and nutrient-poor medium (ANp) which mimics the phagocytic vacuole stress conditions.^33^ LF82 mutants displayed a different proficiency in persisting within MoDCs, both derived from HD and CD patients (Figure 3e). No correlation was observed between intracellular persistence within moDCs and bacterial growth curve in the ANp medium of mutants (Figure S3), thus indicating that ANp formulation did not properly mimic the DC intracellular environment. No correlation was observed either between intracellular bacterial load and IL-23 secretion (Figure 3f-left graph). Indeed, 4 out of the 13 mutants with an impaired ability to promote IL-23 secretion by CD-moDCs, survived within moDCs similarly to the parental LF82 strain (T74.A5, T75.E8, T82.D7, and T96.B8) (Figure 3e). Likewise, 3 out of 9 mutants that triggered a similar amount of IL-23 as LF82 did not persist within CD-moDCs (T31.B12, T52.H10, and T53.A8), thus confirming that the reduced IL-23 secretion by CD-moDCs was not due to a lower intracellular bacterial load at 24 h post infection. Conversely, IL-1β secretion was dependent on intracellular bacterial load exclusively in CD-moDCs (Figure 3f-right graph).

### 3.3. Several AIEC determinants promoted the dysregulated IL-23 hypersecretion by CD-derived moDCs

To identify the mutated genes in the final 13 selected LF82 mutants (Figure 3d-upper graph), and to analyze the effect of these mutations on the expression of the key AIEC pathogenic features, we performed an in depth phenotypic characterization. The DNA sequencing after rescue cloning revealed that the transposon insertions in these 13 selected LF82 mutants occurred within genes involved in a few specific pathways including metabolic processes, stress response, LPS and capsule biosynthesis (Table 1, Figure S4).

**Table 1.** Target gene of the EZ-Tn5-based transposon in LF82 mutant strains.

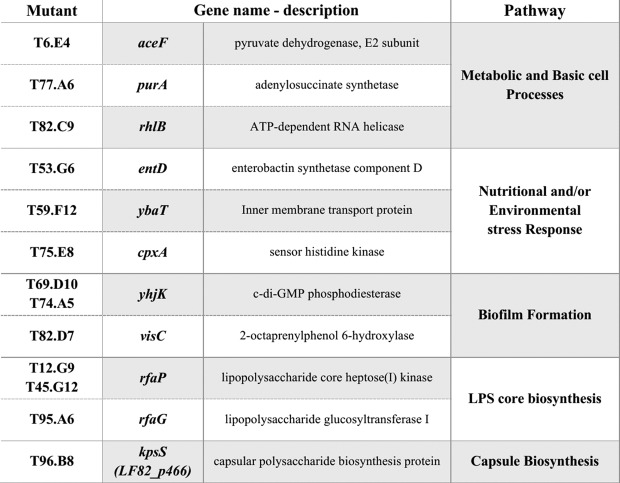

Importantly, 2 out of 3 mutants in pathways involved in biofilm formation (T69.D10 and T74.A5), and 2 out of 3 mutants in pathways involved in the LPS core biosynthesis (T12.G9 and T45.G12) resulted mutated in the same gene, *yhjK* and *rfaP*, respectively, thus confirming the strength of our functional screenings.

**Figure 4.**
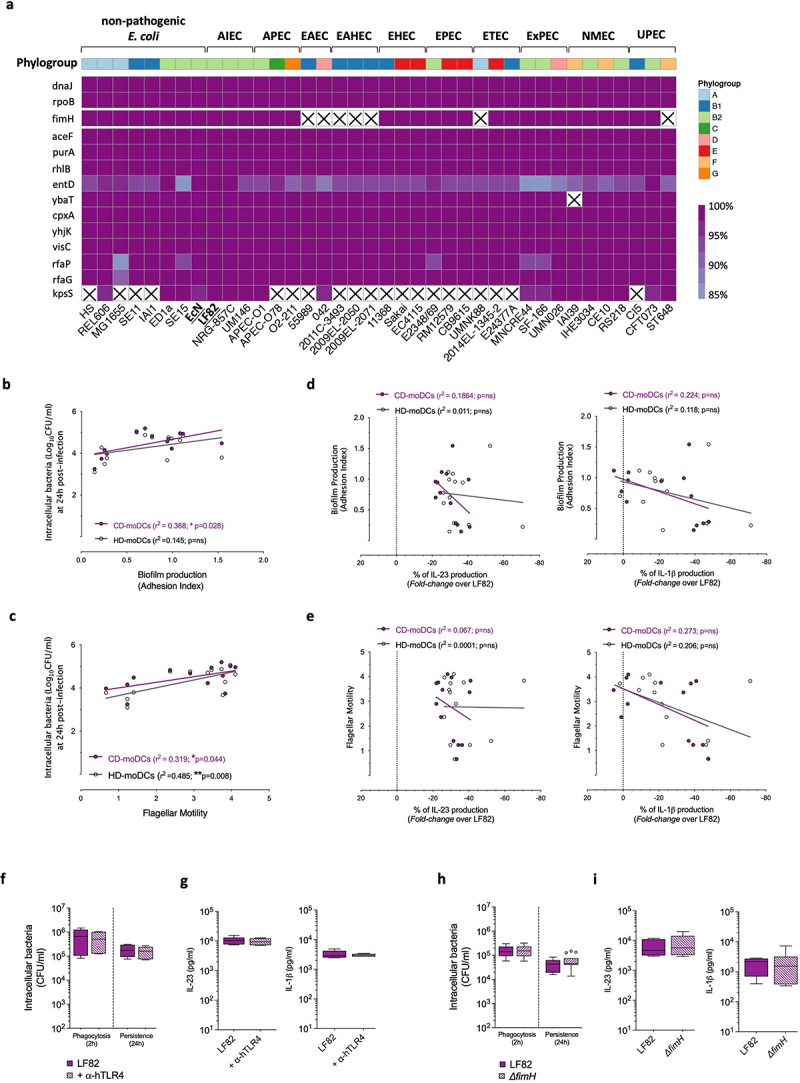
Conservation of selected AIEC-gene targets among different *E. coli* pathotypes and phenotypic characterization of the LF82 mutant strains.

Remarkably, except for *entD* and *kpsS*, which displayed relatively lower levels of conservation and a patchy phylogenetic distribution, all the genes identified by our screening were almost universally present across distinct *E. coli* pathotypes and phylogroups (Figure 4a). These 11 genes showed high levels of sequence conservation, with a pattern that was not dissimilar to the universal single copy marker genes *dnaJ* and *rpoB*. Similar considerations could be applied to *fimH*, one of the most studied AIEC virulence factors,^29,49,50^ which was found to be associated with all the *E. coli* strains included in our analyses (Figure 4a) with the exclusion of EAEC and EAHEC pathotypes, as previously demonstrated.^39^

(a) Heatmap showing presence and conservation (protein sequence identity) of AIEC-target genes (rows) of the 13 LF82 mutants with an impaired ability to stimulate IL-23 secretion by CD-moDCs, among different *E. coli* phylogroups and pathotypes (columns). Columns are organized first by pathotype, namely Adherent-Invasive (AIEC), Avian Pathogenic (APEC), Enteroaggregative (EAEC), Enteroaggregative Hemorrhagic (EAHEC), Enterohemorrhagic (EHEC), Enteropathogenic (EPEC), Enterotoxigenic (ETEC), Extraintestinal Pathogenic (ExPEC), Neonatal Meningitis (NMEC), Uropathogenic (UPEC), and then by phylogroup indicated by the colored squares. Identity levels are represented according to the color gradient indicated by the scale. White barred squares indicate the absence of relative gene in the analyzed bacterial strains. The *fimH*, one of the most studied AIEC virulence factors, is also included in this analysis for comparative purpose, while *rpoJ* and *dnaJ* are used to represent the identity levels and phylogenetic distribution of universal single copy marker genes. Graphical representation of the data by heatmap R library. R version 4.2.1 was used.

Correlation between biofilm formation (b) or flagellar motility (c) of the 13 selected LF82 mutants and their intracellular bacterial load within HD- (white dots) or CD-moDCs (purple dots) at 24 h post-infection. Correlations were determined using Pearson correlation coefficients. R squares and *p* values for each correlation were reported in the graphs.

Correlation between biofilm formation (d) or flagellar motility (e) and fold changes in the percentage of IL-23 (**left graphs**) or IL-1β (**right graphs**) production of each LF82 mutants, as compared to the parental LF82 strain, within HD- (white dots) or CD-moDCs (purple dots) at 24 h post-infection. Correlations were determined using Pearson correlation coefficients. R squares and *p* values for each correlation were reported in the graphs.

(f) Intracellular bacterial load of LF82 within CD-moDCs after 2 h (phagocytosis) or after 24 h of infection (persistence), in the presence or absence of human-TLR4 neutralizing antibody (α-hTLR4; *n* = 3).

(g) Secretion of IL-23 (**left graph**) and IL-1β (**right graph**) by CD-moDCs after 24 h of infection with LF82 in the presence or absence of α-hTLR4 neutralizing antibody (*n* = 3).

(h) Intracellular bacterial load of LF82 or LF82-*ΔfimH* isogenic deletion mutant within CD-moDCs (*n* = 5) after 2 h (phagocytosis) or 24 h of infection (persistence).

(i) Production of IL-23 (**left graph**) and IL-1β (**right graph**) by CD-moDCs after 24 h of infection with LF82 or LF82-*ΔfimH* isogenic deletion mutant (*n* = 5).

(f-i) Data are represented as mean ± SEM of 3–5 independent experiments. Statistical significance was calculated using Mann–Whitney’s test.

Next, we analyzed whether mutations in these 11 genes impacted on the expression of key AIEC pathogenic features, including biofilm formation, motility, and fimbriae expression (Table 2, Figure S5). Phenotypic characterization confirmed the higher proficiency of AIEC to form biofilm as compared to EcN (Table 2) and the distinctive expression of type 1 fimbriae (FimH) by AIEC^31^ (Figure S5A), despite the presence of *fimH* gene both in LF82 and EcN with a 98.67% of protein sequence identity level (Figure 4a). In contrast, both LF82 and EcN resulted curli-positive displaying the “rdar” (for red, dry, and rough) morphology on CR agar plates (Table 2, Figure S5b), consistently with the curli production by most of Enterobacteriaceae.^43^

**Table 2.** Phenotypic characterization of the adhesion determinants production.

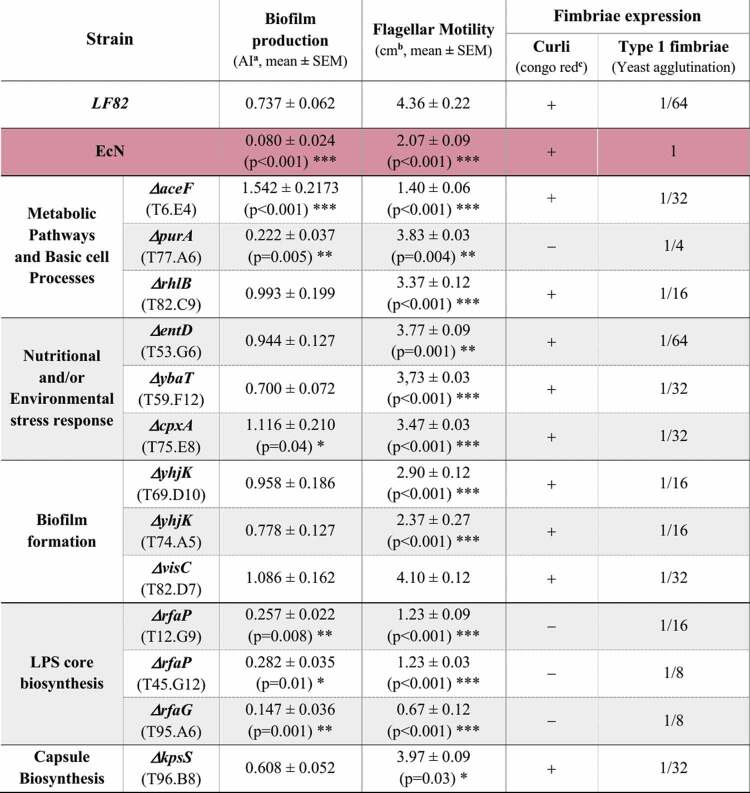

^**a**^Adhesion index, calculated as previously described.^35^ Statistical significance was calculated using Mann-Whitney’s test and reported as *p<0.05, ** *p*<0.01, *** *p*<0.001.

^**b**^Halo diameter (cm) in motility soft agar plates. Statistical significance was calculated using Mann-Whitney’s test and reported as *p<0.05, ** *p*<0.01, *** *p*<0.001.

^**c**^+ indicates “rdar” (for red, dry, and rough) phenotype, – for white colonies on congo red (CR) agar plates.

The phenotypic analysis of the 13 selected mutants displayed a positive correlation both between AIEC intracellular persistence and biofilm production (Figure 4b), and flagellar motility (Figure 4c). Instead, neither biofilm production nor flagellar motility correlated with IL-23 or IL-1β secretion by DCs (Figure 4d,e) thus indicating that AIEC adhesion determinants are not relevant for this type of inflammatory response by DCs. Notably, among mutants promoting the lowest IL-23 secretion by CD-moDCs, even lower than EcN (Figure 3D-upper graph), we found mutants in the LPS structure (Δ*rfaP* and Δ*rfaG)* and the Δ*purA* mutant, which did not produce curli fimbriae and displayed the highest reduction in FimH production compared to the parental strain LF82 (Table 2, Figure S5). These results, together with the evidence that several pathogenic *E. coli* strains trigger mucosal inflammation via fimbriae-dependent TLR4 activation,^51,52^ led us to investigate whether also the IL-23 hypersecretion by CD-moDCs may be TLR4-dependent. Blockade of the TLR4 biological activity with neutralizing antibodies did not affect neither the LF82 phagocytosis/persistence within CD-moDCs (Figure 4f), nor IL-23/IL-1β secretion (Figure 4g). Similarly, also the deletion of *fimH*, a potent TLR4 agonist that promotes inflammation in an LPS-independent manner,^53^ did not hamper LF82 internalization or persistence within CD-moDCs (Figure 4h) and neither triggered a lower inflammatory response (Figure 4i), thus indicating that fimbriae-LPS/TLR4-dependent signaling pathway does not play a major role in the AIEC intracellular persistence or in the inflammatory response of DCs to AIEC infection.

Taken together, these results indicated that AIEC determinants implicated in triggering IL-23 release and survival within DCs are different from AIEC adhesion determinants promoting adhesion and invasion of intestinal epithelial cells.^29,42^

### 3.4. Mutations in an inner membrane protein and in the LPS structure hamper the AIEC-dependent transdifferentiation of cTh17 into pathogenic IFNγ-producing pTh17 cells

To functionally validate the link between the absence of specific AIEC determinants and the transdifferentiation of cTh17 into pTh17 cells, we screened our selected LF82 mutants with the antigen-specific assay.

**Figure 5.**
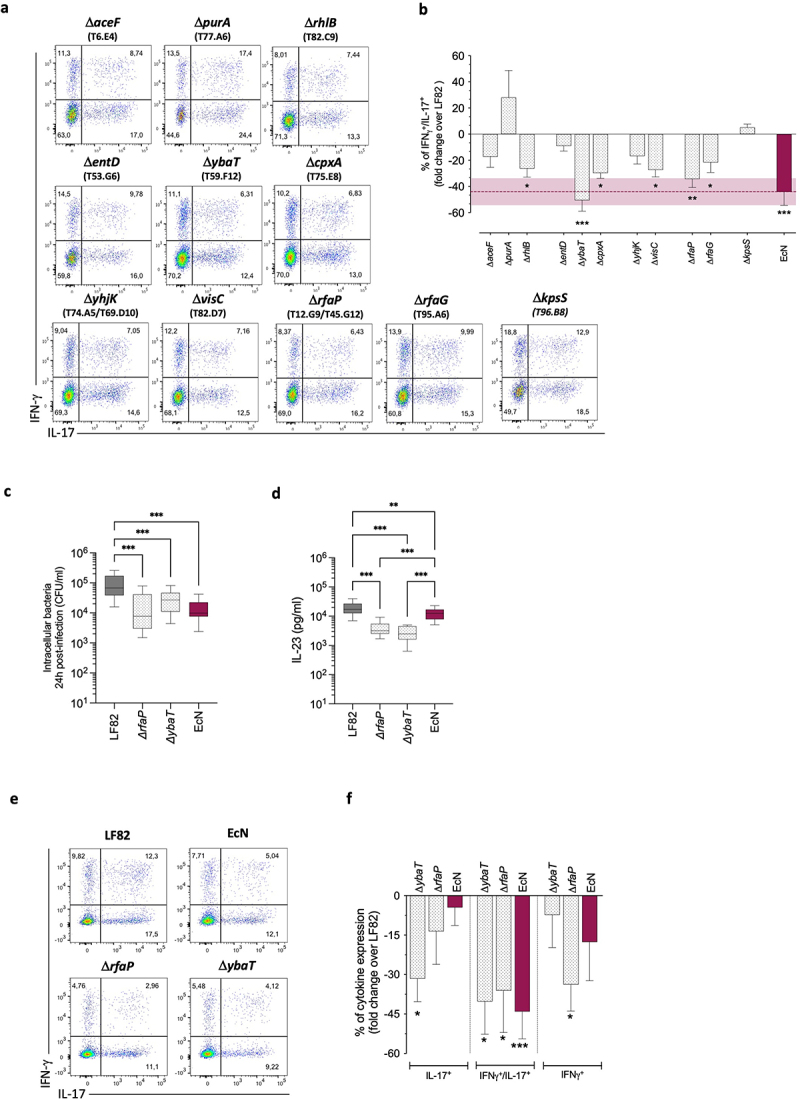
Deletion of the AIEC-determinants*ybaT* or *rfaP* drastically impaired the transdifferentiation of cTh17 cells into pTh17 cells.

Our results revealed that Δ*aceF* (T6.E4), Δ*purA* (T77.A6), Δ*entD* (T53.G6), Δ*yhjK* (T74.A5; T69.D10), and Δ*kpsS* (T96.B8) mutants, albeit exhibited a significant reduction in IL-23 secretion by CD-moDCs (Figure 3d-upper graph) and, except for Δ*yhjK* and Δ*kpsS*, also a defective intracellular persistence (Figure 3e), did not significantly impair the IFNγ/IL-17 co-expression by cTh17 cells as compared to LF82 (Figure 5a,b), thus not representing the most interesting candidate targets.

(a) One representative experiment out of four showing the gating strategy to identify antigen-dependent pTh17 transdifferentiation after 10 d of cTh17 cell stimulation with autologous moDCs infected with AIEC-LF82 or indicated LF82 mutant strains is shown.

(b) Fold-change values of the percentages of IFNγ^+^/IL-17^+^ co-expression on peripheral CD40L^+^cTh17 cells after 10 d of co-culture with autologous CD-moDCs infected with indicated mutants. Data are represented as mean percentage (fold-change over LF82) ± SEM of at least four independent experiments. Statistical significance was calculated using one-way ANOVA and reported as *p < 0.05, ** *p* < 0.01, *** *p* < 0.001.

Intracellular bacterial load after 24 h of infection (c) and IL-23 production (d) by CD-moDCs infected with LF82, indicated LF82-isogenic deletion mutants, and probiotic EcN strain (*n* = 6). Data are represented as mean percentage (fold-change over LF82) ± SEM of six independent experiments. Statistical significance was calculated using one-way ANOVA and reported as *p < 0.05, ** *p* < 0.01, *** *p* < 0.001.

(e) One representative experiment out of three, showing the gating strategy to identify antigen-dependent pTh17 transdifferentiation in response to LF82-isogenic deletion mutants, is shown.

(f) Fold-change values of the percentages of single IFNγ^+^, IL-17^+^ expression and IFNγ^+^/IL-17^+^ co-expression on peripheral CD40L^+^cTh17 cells after 10 d of co-culture with autologous CD-derived moDCs infected with indicated LF82-isogenic deletion mutants. Data are represented as mean percentage (fold-change over LF82) ± SEM of at least three independent experiments. Statistical significance was calculated using one-way ANOVA and reported as *p < 0.05, ** *p* < 0.01, *** *p* < 0.001.

On the contrary, six mutants significantly reduced the IFNγ/IL-17 co-expression and differently modulated the single expression of IL-17 or IFNγ by stimulated cTh17 cells compared to the parental LF82 strain (Figure 5a,b and S6a). Among them, Δ*ybaT* (T59.F12) and in a lesser extend Δ*rfaP* (T12.G9 and T45.G12) mutants drastically impaired the skewing of cTh17 into pTh17 cells (Figure 5b), inducing similar IFNγ/IL-17 co-expression levels as observed in response to the probiotic EcN strain (Figure 2b), or after prolonged IL-23 neutralization (Figure 2g-right graph). None of these strains affected the viability, fitness, or activation of cTh17 cells (Figure S6b).

Finally, to exclude putative polar effects of transposon insertion in our mutants, and to validate whether the AIEC virulence determinants identified through the screenings of our library were effective candidate targets to hinder pTh17 cell transdifferentiation, we generated individual isogenic deletion mutants in *rfaP* and *ybaT* genes. Our data strongly confirmed that the absence of *rfaP* or *ybaT* genes in LF82 led to a compromised intracellular persistence within CD-moDCs (Figure 5c), together with a significant reduction in IL-23 secretion (Figure 5d) and, especially, to a drastic impairment of pTh17 cell generation (Figure 5e,f).

In summary, our findings indicated that the transdifferentiation of pTh17 cell is strongly driven in CD patients by some AIEC determinants, generated within antigen processing compartments of DCs, and presented to cTh17 cells together with very high IL-23 levels selectively secreted by AIEC-infected DCs. Therefore, targeting the AIEC virulence determinants specifically involved in triggering IL-23 signaling and in the harmful transdifferentiation of pTh17 cells, as those identified in this study, could represent a promising and unprecedented therapeutic strategy to prevent chronic intestinal inflammation in CD patients.

## 4. Discussion

The chronic and progressive nature of the CD is correlated with an exaggerated mucosal adaptive inflammatory response against luminal bacterial antigens, with the AIEC pathotype proposed as one of the main triggers.^25,26^ Although the strong association between AIEC and CD gut dysbiosis has been extensively demonstrated,^25,54^ comparative genomics analysis failed to identify a characteristic genomic signature profile for AIEC distinguishing it from other pathogenic or commensal *E. coli* strains. As a result, AIEC cannot be selectively targeted with the common microbial-based or microbiota-targeted therapies.^55^

An increasing body of evidence points to a crucial and prominent role in the pathogenesis of CD for IL-23-dependent activation of pathogenic intestinal resident IFNγ-producing Th17 cells (pTh17 cells) in CD patients.^24,56^ We recently demonstrated that pathogenic intestinal resident IFNγ-producing pTh17 cells isolated from CD, but not from UC patients, are selectively activated by AIEC strains and are specifically decreased following anti-IL-23 treatment.^24^ This work paved the way in defining a role for AIEC in triggering IL-23 secretion and pTh17 cell activation without, however, elucidating the underlying molecular mechanisms.

Most of the therapeutic strategies in CD are based on systemic immunomodulation or targeting pro-inflammatory cytokines involved in the activation of mucosal T_RM_ cells, such as antibodies against TNFα, IL-12 and/or IL-23, or against integrin α4β7.^57^ In this context, however, the relationship between AIEC and the dysregulated activation of IL-23/pTh17 axis in CD patients has never been fully characterized.

AIEC virulence is mainly defined at the phenotypic level for its ability to adhere and invade intestinal epithelial cells (IECs), together with its proficiency in persisting within macrophages.^30^ Several AIEC virulence determinants have been described to play a crucial role in these processes, promoting a sustained inflammatory response, and thus contributing to the chronic intestinal inflammation in CD.^49,50^ For example, intramacrophage replication of AIEC strongly promotes mucosal inflammation through the secretion of very high TNFα levels.^30^ Here, instead, we observed that AIEC intramacrophage persistence did not promote neither IL-23 nor very high levels of IL-1β, and, especially, that AIEC-infected macrophages completely failed to induce pTh17 cells transdifferentiation from protective cTh17 cells. These results are in line with the clinical evidence reporting that anti-TNFα drugs hamper AIEC replication within macrophages^58^ but do not affect the IL-23/IL-17 axis.^59^ In contrast, DCs derived from CD patients secreted very high IL-23 levels in response to AIEC strains and, importantly, also in response to the probiotic EcN strain compared to DCs isolated from healthy subjects. These results not only confirm the genetic predisposition of CD patients to an overwhelming mucosal inflammatory response^1^ but also imply that some probiotics could be ineffective or even detrimental for CD patients.^35,60^ EcN strain harbors some key virulence genes commonly associated with pathogenic phenotypes in *E. coli*,^61^ including some LF82 genes with immunostimulatory potential.^24^ Nevertheless, the EcN-DCs interaction did not promote pTh17 cell generation, suggesting that the expression of certain bacterial antigens leading to the transdifferentiation of pTh17 cells might be selectively processed within CD-derived DCs during AIEC infection.

In this work, we aimed to identify and functionally characterize the AIEC virulence determinants specifically implicated in pTh17 cell differentiation. By generating and screening a library of more than 10.000 AIEC transposon insertion mutants, we demonstrated that the AIEC molecular determinants triggering the IL-23 hypersecretion by CD-DCs and promoting pTh17 cell generation, were distinct from those associated with the AIEC intracellular persistence and the exaggerated inflammatory response in macrophages and IECs. Indeed, among AIEC mutants defective in triggering IL-23 we did not find mutants in virulence genes commonly associated with AIEC pathogenicity like *ibeA*, *htrA*, *fimH*, *chiA*.^33,49,50^ The screenings of our AIEC mutant library revealed instead that the AIEC-dependent IL-23 hypersecretion by CD-derived DCs was linked to a restricted subset of pathways, mainly involved in metabolic and stress response processes, biofilm formation, LPS and capsule biosynthesis. Interestingly, most of the genes identified by our screenings displayed a broad phylogenetic distribution within *E. coli* and are consistently found in the *E. coli* genomes belonging to different pathotypes and phylogroups, with levels of conservation comparable to those of some universal single copy number genes. Moreover, if this observation confirms that AIEC-dependent intestinal inflammation in CD relies on the complex interplay of several factors (i.e., genetic, environmental, and immunological) and not exclusively on the AIEC virulence determinants, it remains unclear why these genes do not confer a selective advantage in colonizing the ileal mucosa of CD patients and promoting chronic inflammation also to other *E. coli* pathotypes.

Several works propose a direct correlation between the expression of specific bacterial virulence determinants (i.e., biofilm formation, fimbriae/adhesins expression) and the AIEC proficiency to invade and persist within host cells like IECs and macrophages.^50,62^ In contrast, our results clearly demonstrated that mutants defective in persisting within DCs and triggering lower IL-23 secretion displayed both a biofilm-, curli-, and fimbriae-producing phenotype (i.e., Δ*aceF, ΔrhlB*) and a non-producing phenotype (i.e., Δ*purA* and LPS mutants). Moreover, deletion in LF82 of the FimH adhesin, one of the best characterized AIEC virulence factors,^29,49,50^ whose CEACAM6 receptor is upregulated in IECs of CD patients^31^ and expressed on myeloid cells, did not affect neither the uptake and survival of AIEC within DCs nor the inflammatory response. Therefore, our data indicated that the AIEC virulence determinants promoting invasion, intracellular replication, and the inflammatory response in IECs and macrophages are not involved in the IL-23 hypersecretion and pTh17 cell generation which instead rely on the functions of dendritic cells. Epithelial infiltrating DCs can indeed directly detect bacteria across the mucosal epithelium,^63^ leading to the secretion of high IL-23 levels and, especially in CD patients where AIEC is highly enriched,^26^ to pTh17 cell transdifferentiation. Therefore, blocking the interaction between AIEC and intestinal epithelial cells may not prevent DC-dependent activation of AIEC-specific pTh17 cells.

Conversely, our results outlined a new crucial point in the knowledge of AIEC virulence, defining the AIEC determinants specifically involved in triggering IL-23 hypersecretion and in priming pTh17 cells in CD. Our functional assays identified the LPS core heptose(I) kinase *rfaP*, and the inner membrane transport protein *ybaT*, as two key AIEC virulence determinants directly involved in the skewing of pTh17 cells from protective cTh17 cells. Interestingly, 2 mutants were found in distinct genes of the *rfa* operon, Δ*rfaP* and Δ*rfaG*, which promoted both a significant lower IL-23 secretion and a strong impairment in the LF82 intracellular persistence, but mutation in *rfaP* displayed a higher inhibition of pTh17 cell transdifferentiation as compared to mutation in *rfaG*. These differences, together with the different phenotype of Δ*rfaP* and Δ*rfaG* mutants compared to the parental LF82 strain, both in terms of biofilm production, motility, and fimbriae expression, are most probably linked to the pleiotropic effects of LPS-genes mutations on the outer membrane protein properties as previously described by Pagnout and colleagues.^64^ Indeed, we exclude that the lower IL-23 secretion, and the impaired intracellular persistence of Δ*rfaP* and Δ*rfaG* mutants, could be exclusively attributed to a truncated LPS structure since the TLR4 blockade – which has been proposed moreover as a promising therapeutic approach because extensively upregulated in CD^65–^ hampered neither the uptake/intracellular persistence nor the inflammatory response of infected CD-derived DCs.

In this regard, Guttsches and colleagues reported that the truncated LPS structure of the probiotic EcN strain only partially explains its loss of pathogenicity, since LPS extracted from EcN has lower immunomodulatory effects and anti-inflammatory activity than an EcN lysate. Therefore, they proposed that the EcN surface polysaccharide structure, along with other undefined bacterial determinants, are crucial for the protective immunomodulatory effects of this probiotic strain.^66^

We speculate that the absence of *rfaP*, which leads to a modification in the LPS inner core due to the lack of phosphorylation of the first heptose added to KDO2-lipid A structure, results in an altered structure and stability of the outer membrane. Consequently, during the intracellular processing of the Δ*rfaP* mutant, it is possible that the altered membrane structure leads to the generation of different AIEC antigens compared to those generated from the parental AIEC LF82 strain, which fail to generate pTh17 cell when loaded on MHC-II of DCs.

Similarly, mutations in *ybaT*, encoding an amino acid permease involved in the glutamate-dependent acid tolerance system within the gene cluster implicated in copper (Cu) tolerance, resulted in a compromised release of IL-23 and abrogated pTh17 cell generation.

It is known that enteric pathogens, like AIEC, respond to high Cu levels and low pH as those encountered in the small intestine and within phagolysosomes through Cu efflux pumps. In other pathogenic *E. coli* strains, deletion of *ybaT* leads to loss of protection against acid stress and Cu accumulation, resulting in a diminished ability of bacteria to survive under these stress conditions.^67^ Moreover, it has been demonstrated that the adaptive response of bacteria to acid stress leads to changes in the membrane permeability and modification in the lipid bilayer.^68^

Therefore, it is likely that the absence of *ybaT* in the inner membrane of AIEC leads to an altered membrane composition or stability, which in turn promotes a different antigenic intracellular processing failing to generate AIEC antigens capable of inducing the skewing of cTh17 to pTh17 cells.

Importantly, in contrast to some virulence factors like FimH which is absent in other pathogenic *E. coli* strains, *YbaT* is ubiquitously found across all *E. coli* pathotypes, also showing a narrow phylogenetic distribution across Enterobacteriales (observed in 8/522 non-*E. coli* genomes according to the orthoDB database)^69^ and therefore, could represent an ideal therapeutic target.

Although further studies will be needed to specifically define which AIEC antigens are altered or no longer generated during the intracellular processing of these mutants, *rfaP* and *ybaT* undoubtedly represent promising and unprecedented candidate therapeutic targets to prevent chronic intestinal inflammation in CD patients. Indeed, our results demonstrated that the targeting of *ybaT* and *rfaP* induced a drastic reduction both in AIEC intracellular persistence and IL-23 secretion by CD-DCs and, most importantly, inhibited the skewing of cTh17 cells toward the pTh17 pathogenic phenotype without affecting the viability and function of protective Th17 cells. This point is of particular significance if we consider that current therapeutic strategies aimed to block IL-23 result in a high remission rate in CD patients, but neither completely abolish T_RM_ pTh17 cells^24^ nor prevent further microbial-dependent production of IL-23. Accordingly, only a continuous IL-23 blockade significantly reduced the in vitro AIEC-dependent pTh17 cell differentiation. This result depends on the high proficiency of AIEC in persisting for a long time within DCs, and thus resulting in a chronic hypersecretion of IL-23. In this regard, further studies correlating mucosal AIEC abundance in CD patients with clinical response to anti-IL-23 treatment could be of crucial importance for understanding disease progression as well response rates to therapies. Moreover, besides its contributing role in the pTh17 cell induction and in CD pathogenesis, IL-23 also plays important homeostatic functions by regulating innate lymphoid cells (ILC) or enhancing intestinal barrier mechanism by promoting the release of antimicrobial peptides.^70^ Therefore, targeting upstream the bacterial stimuli selectively triggering the IL-23 overproduction could be a turning point in the treatment of chronic intestinal inflammation in CD.

In conclusion, here we identified two AIEC virulence determinants directly implicated in triggering IL-23 hypersecretion and IFNγ-producing pTh17 cell generation, two immunological pathways highly dysregulated in CD patients. Particularly, *rfaP* and *ybaT* represent paramount bacterial targets for the development of more efficient therapeutic strategies, which may have the great advantage of selectively preventing the activation of the IL-23/pTh17 axis leaving protective immune cells unaffected.

## Supplementary Material

Supplemental Material

## Data and material availability

All the data required to evaluate the conclusions presented in this paper are included within the paper and supplementary material. All LF82 mutant strains used in the current study are available from the lead contact with a completed Materials Transfer Agreement.

## Dedication

This paper is dedicated to the memory of our dear colleague Nicolas Barnich who recently passed away. His untimely departure is a profound loss to the scientific community and to all who had the privilege of knowing him. Prof. Barnich’s contributions to this work, and to the field at large, were invaluable. His dedication, intellectual rigor, and passion for research were evident in every project he undertook. He was not only a brilliant scientist, a dedicated and thorough collaborator, but also a generous mentor and a cherished friend. We will deeply miss him.”
