# Supplementary material for: AIEC-dependent pathogenic Th17 cell transdifferentiation in Crohn’s disease is suppressed by rfaP and ybaT deletion
Source: Gut Microbes. 2024 Jul 29;16(1):2380064. doi: 10.1080/19490976.2024.2380064 (PMC11290758; doi:10.1080/19490976.2024.2380064)
Supplement: Supplemental Material [file KGMI_A_2380064_SM7770.zip › Leccese_G_et_al_Supplementary_materials_WITH_REFERENCES_.pptx]

## Slide 1
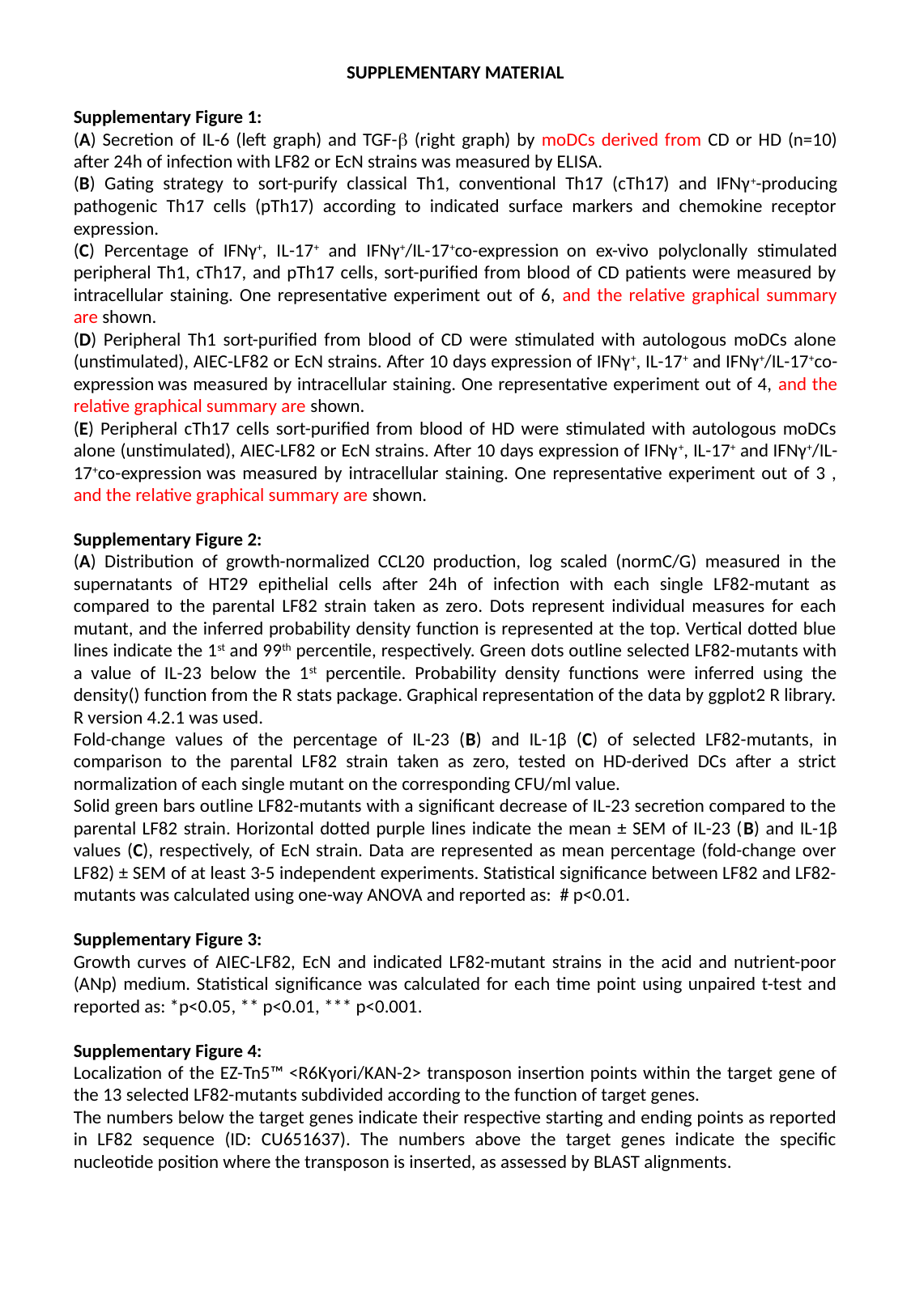

SUPPLEMENTARY MATERIAL
Supplementary Figure 1:
(A) Secretion of IL-6 (left graph) and TGF-β (right graph) by moDCs derived from CD or HD (n=10) after 24h of infection with LF82 or EcN strains was measured by ELISA.
(B) Gating strategy to sort-purify classical Th1, conventional Th17 (cTh17) and IFNγ+-producing pathogenic Th17 cells (pTh17) according to indicated surface markers and chemokine receptor expression.
(C) Percentage of IFNγ+, IL-17+ and IFNγ+/IL-17+co-expression on ex-vivo polyclonally stimulated peripheral Th1, cTh17, and pTh17 cells, sort-purified from blood of CD patients were measured by intracellular staining. One representative experiment out of 6, and the relative graphical summary are shown.
(D) Peripheral Th1 sort-purified from blood of CD were stimulated with autologous moDCs alone (unstimulated), AIEC-LF82 or EcN strains. After 10 days expression of IFNγ+, IL-17+ and IFNγ+/IL-17+co-expression was measured by intracellular staining. One representative experiment out of 4, and the relative graphical summary are shown.
(E) Peripheral cTh17 cells sort-purified from blood of HD were stimulated with autologous moDCs alone (unstimulated), AIEC-LF82 or EcN strains. After 10 days expression of IFNγ+, IL-17+ and IFNγ+/IL-17+co-expression was measured by intracellular staining. One representative experiment out of 3 , and the relative graphical summary are shown.
Supplementary Figure 2:
(A) Distribution of growth-normalized CCL20 production, log scaled (normC/G) measured in the supernatants of HT29 epithelial cells after 24h of infection with each single LF82-mutant as compared to the parental LF82 strain taken as zero. Dots represent individual measures for each mutant, and the inferred probability density function is represented at the top. Vertical dotted blue lines indicate the 1st and 99th percentile, respectively. Green dots outline selected LF82-mutants with a value of IL-23 below the 1st percentile. Probability density functions were inferred using the density() function from the R stats package. Graphical representation of the data by ggplot2 R library. R version 4.2.1 was used.
Fold-change values of the percentage of IL-23 (B) and IL-1β (C) of selected LF82-mutants, in comparison to the parental LF82 strain taken as zero, tested on HD-derived DCs after a strict normalization of each single mutant on the corresponding CFU/ml value.
Solid green bars outline LF82-mutants with a significant decrease of IL-23 secretion compared to the parental LF82 strain. Horizontal dotted purple lines indicate the mean ± SEM of IL-23 (B) and IL-1β values (C), respectively, of EcN strain. Data are represented as mean percentage (fold-change over LF82) ± SEM of at least 3-5 independent experiments. Statistical significance between LF82 and LF82-mutants was calculated using one-way ANOVA and reported as: # p<0.01.
Supplementary Figure 3:
Growth curves of AIEC-LF82, EcN and indicated LF82-mutant strains in the acid and nutrient-poor (ANp) medium. Statistical significance was calculated for each time point using unpaired t-test and reported as: *p<0.05, ** p<0.01, *** p<0.001.
Supplementary Figure 4:
Localization of the EZ-Tn5™ <R6Kγori/KAN-2> transposon insertion points within the target gene of the 13 selected LF82-mutants subdivided according to the function of target genes.
The numbers below the target genes indicate their respective starting and ending points as reported in LF82 sequence (ID: CU651637). The numbers above the target genes indicate the specific nucleotide position where the transposon is inserted, as assessed by BLAST alignments.

## Slide 2
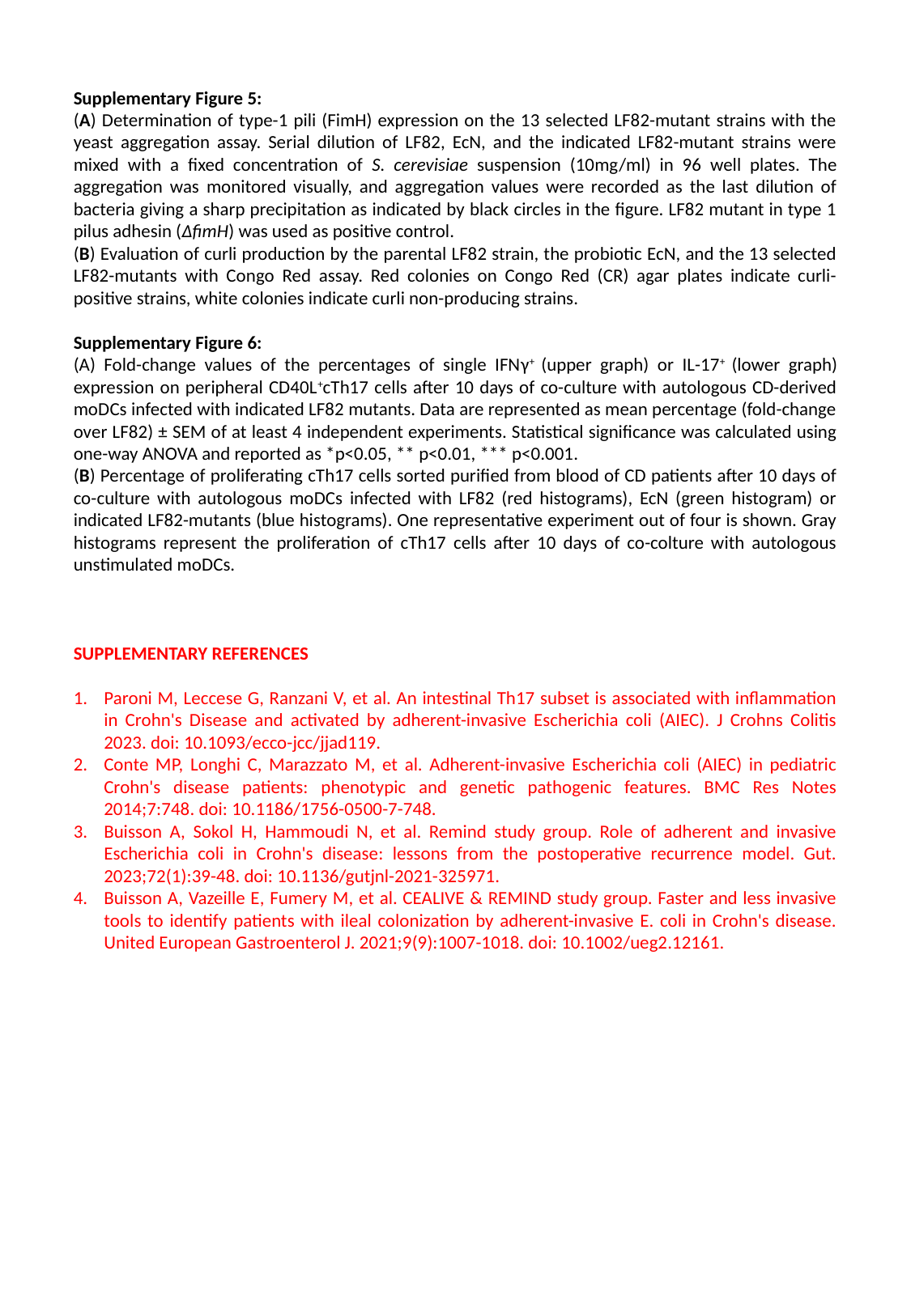

Supplementary Figure 5:
(A) Determination of type-1 pili (FimH) expression on the 13 selected LF82-mutant strains with the yeast aggregation assay. Serial dilution of LF82, EcN, and the indicated LF82-mutant strains were mixed with a fixed concentration of S. cerevisiae suspension (10mg/ml) in 96 well plates. The aggregation was monitored visually, and aggregation values were recorded as the last dilution of bacteria giving a sharp precipitation as indicated by black circles in the figure. LF82 mutant in type 1 pilus adhesin (ΔfimH) was used as positive control.
(B) Evaluation of curli production by the parental LF82 strain, the probiotic EcN, and the 13 selected LF82-mutants with Congo Red assay. Red colonies on Congo Red (CR) agar plates indicate curli-positive strains, white colonies indicate curli non-producing strains.
Supplementary Figure 6:
(A) Fold-change values of the percentages of single IFNγ+ (upper graph) or IL-17+ (lower graph) expression on peripheral CD40L+cTh17 cells after 10 days of co-culture with autologous CD-derived moDCs infected with indicated LF82 mutants. Data are represented as mean percentage (fold-change over LF82) ± SEM of at least 4 independent experiments. Statistical significance was calculated using one-way ANOVA and reported as *p<0.05, ** p<0.01, *** p<0.001.
(B) Percentage of proliferating cTh17 cells sorted purified from blood of CD patients after 10 days of co-culture with autologous moDCs infected with LF82 (red histograms), EcN (green histogram) or indicated LF82-mutants (blue histograms). One representative experiment out of four is shown. Gray histograms represent the proliferation of cTh17 cells after 10 days of co-colture with autologous unstimulated moDCs.
SUPPLEMENTARY REFERENCES
Paroni M, Leccese G, Ranzani V, et al. An intestinal Th17 subset is associated with inflammation in Crohn's Disease and activated by adherent-invasive Escherichia coli (AIEC). J Crohns Colitis 2023. doi: 10.1093/ecco-jcc/jjad119.
Conte MP, Longhi C, Marazzato M, et al. Adherent-invasive Escherichia coli (AIEC) in pediatric Crohn's disease patients: phenotypic and genetic pathogenic features. BMC Res Notes 2014;7:748. doi: 10.1186/1756-0500-7-748.
Buisson A, Sokol H, Hammoudi N, et al. Remind study group. Role of adherent and invasive Escherichia coli in Crohn's disease: lessons from the postoperative recurrence model. Gut. 2023;72(1):39-48. doi: 10.1136/gutjnl-2021-325971.
Buisson A, Vazeille E, Fumery M, et al. CEALIVE & REMIND study group. Faster and less invasive tools to identify patients with ileal colonization by adherent-invasive E. coli in Crohn's disease. United European Gastroenterol J. 2021;9(9):1007-1018. doi: 10.1002/ueg2.12161.

## Slide 3
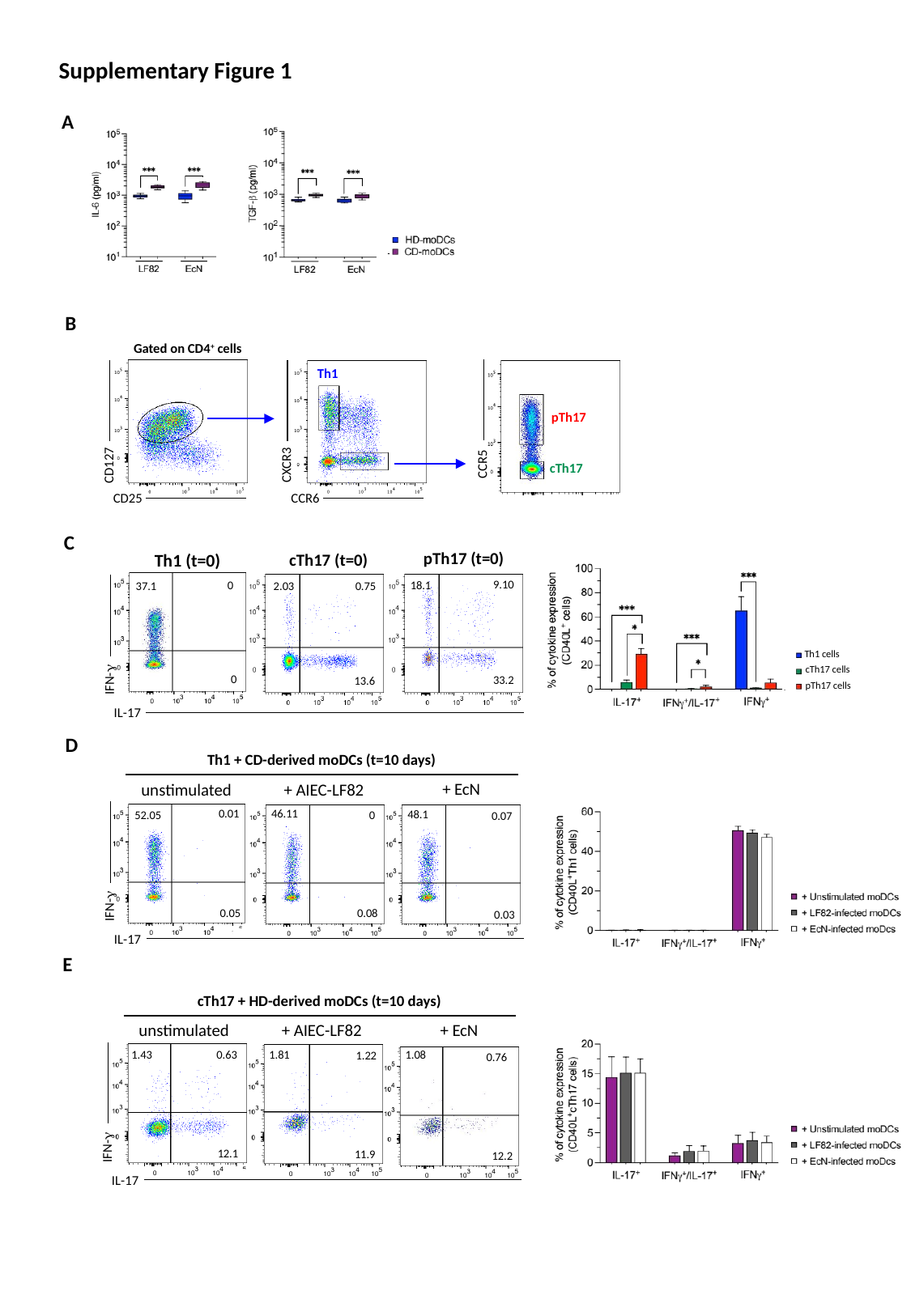

Supplementary Figure 1
A
B
Gated on CD4+ cells
Th1
CD127
CD25
CXCR3
CCR6
pTh17
CCR5
cTh17
C
pTh17 (t=0)
cTh17 (t=0)
Th1 (t=0)
9.10
18.1
33.2
0
0.75
2.03
13.6
37.1
IFN-g
IL-17
Th1 cells
cTh17 cells
0
pTh17 cells
D
Th1 + CD-derived moDCs (t=10 days)
+ EcN
+ AIEC-LF82
unstimulated
46.11
0.01
48.1
52.05
0
0.07
0.05
0.08
0.03
IFN-g
IL-17
E
cTh17 + HD-derived moDCs (t=10 days)
+ EcN
+ AIEC-LF82
unstimulated
1.81
0.63
1.08
1.43
1.22
0.76
12.1
11.9
12.2
IFN-g
IL-17

## Slide 4
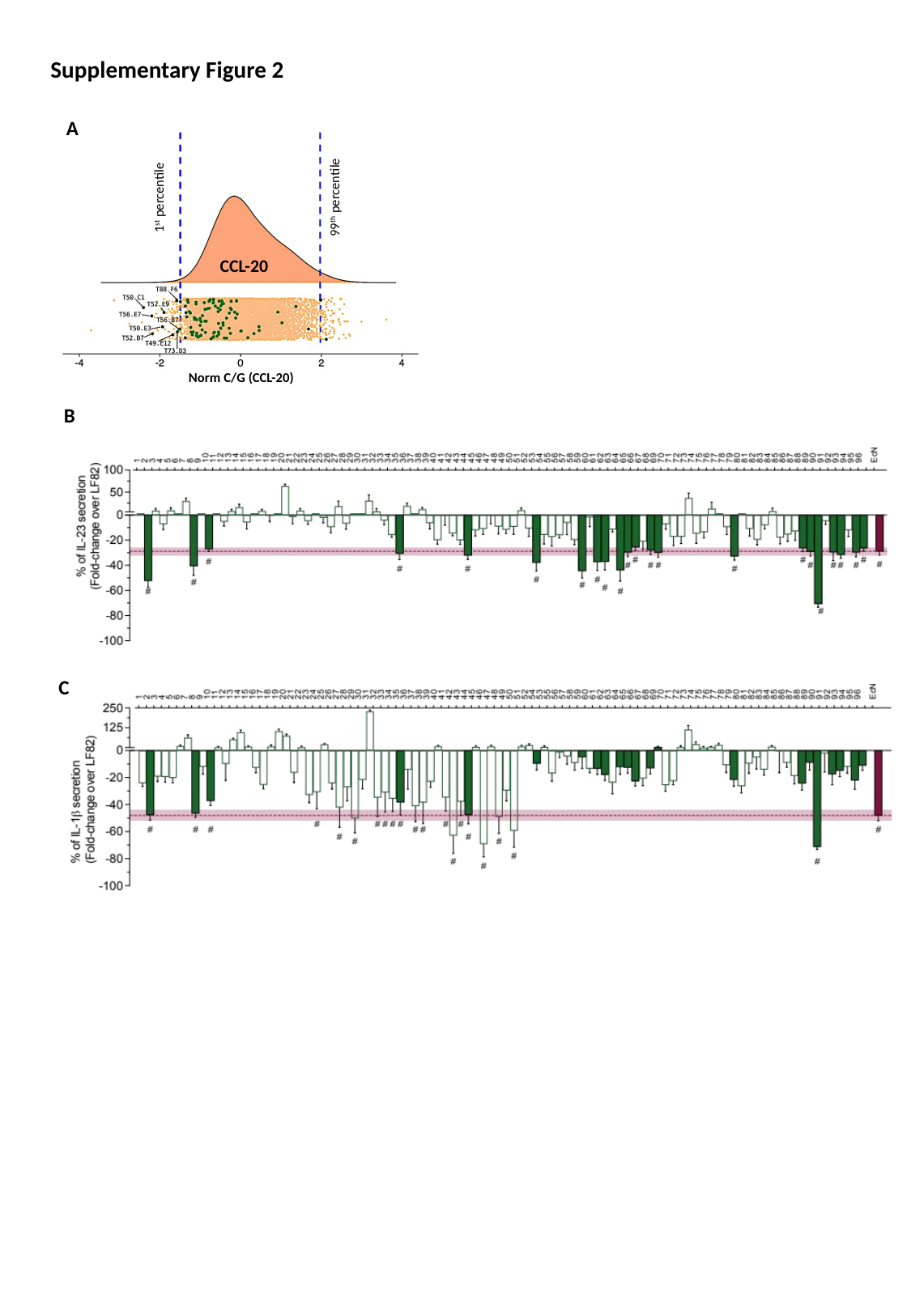

Supplementary Figure 2
A
1st percentile
99th percentile
CCL-20
Norm C/G (CCL-20)
B
C

## Slide 5
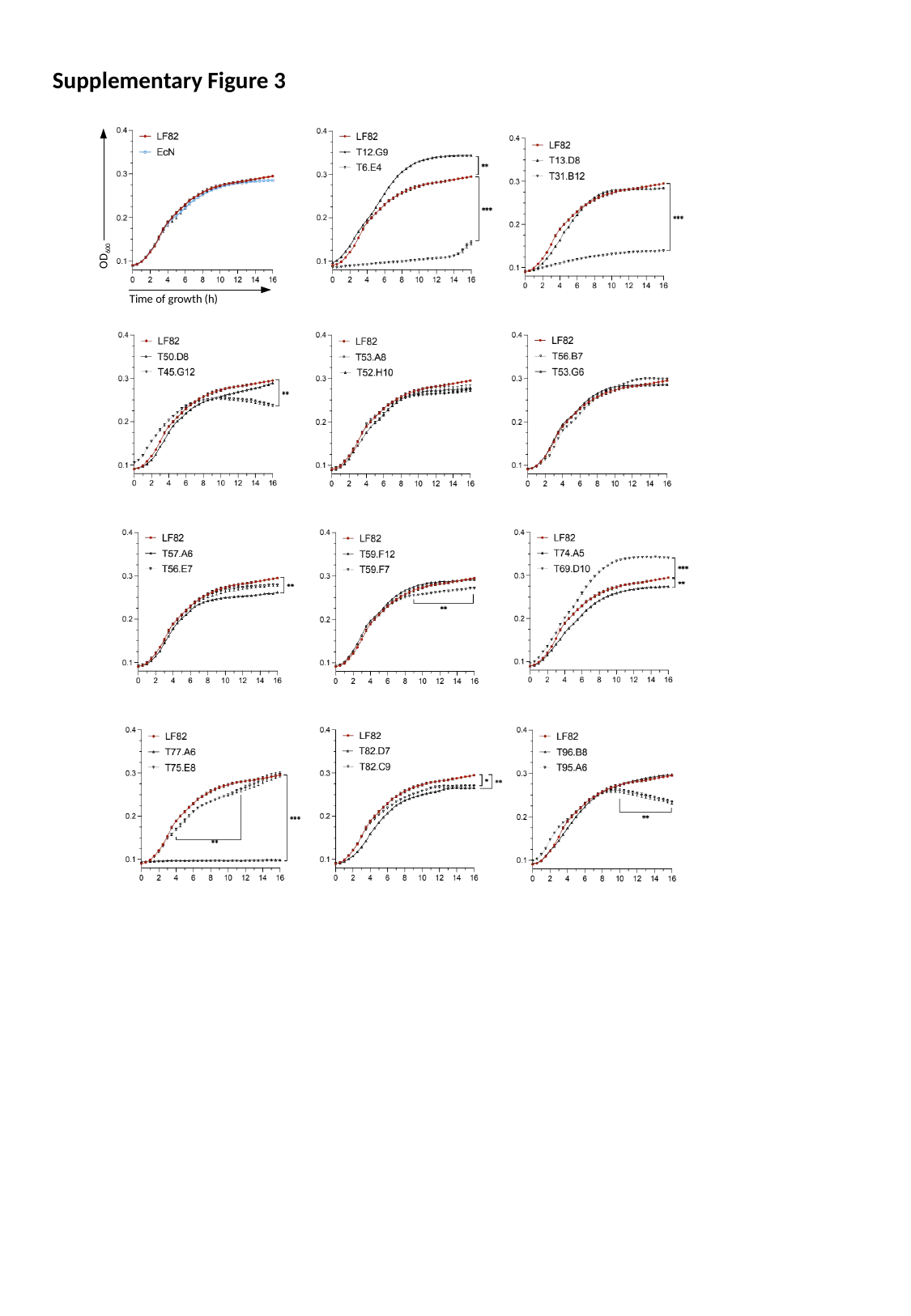

Supplementary Figure 3
OD600
Time of growth (h)

## Slide 6
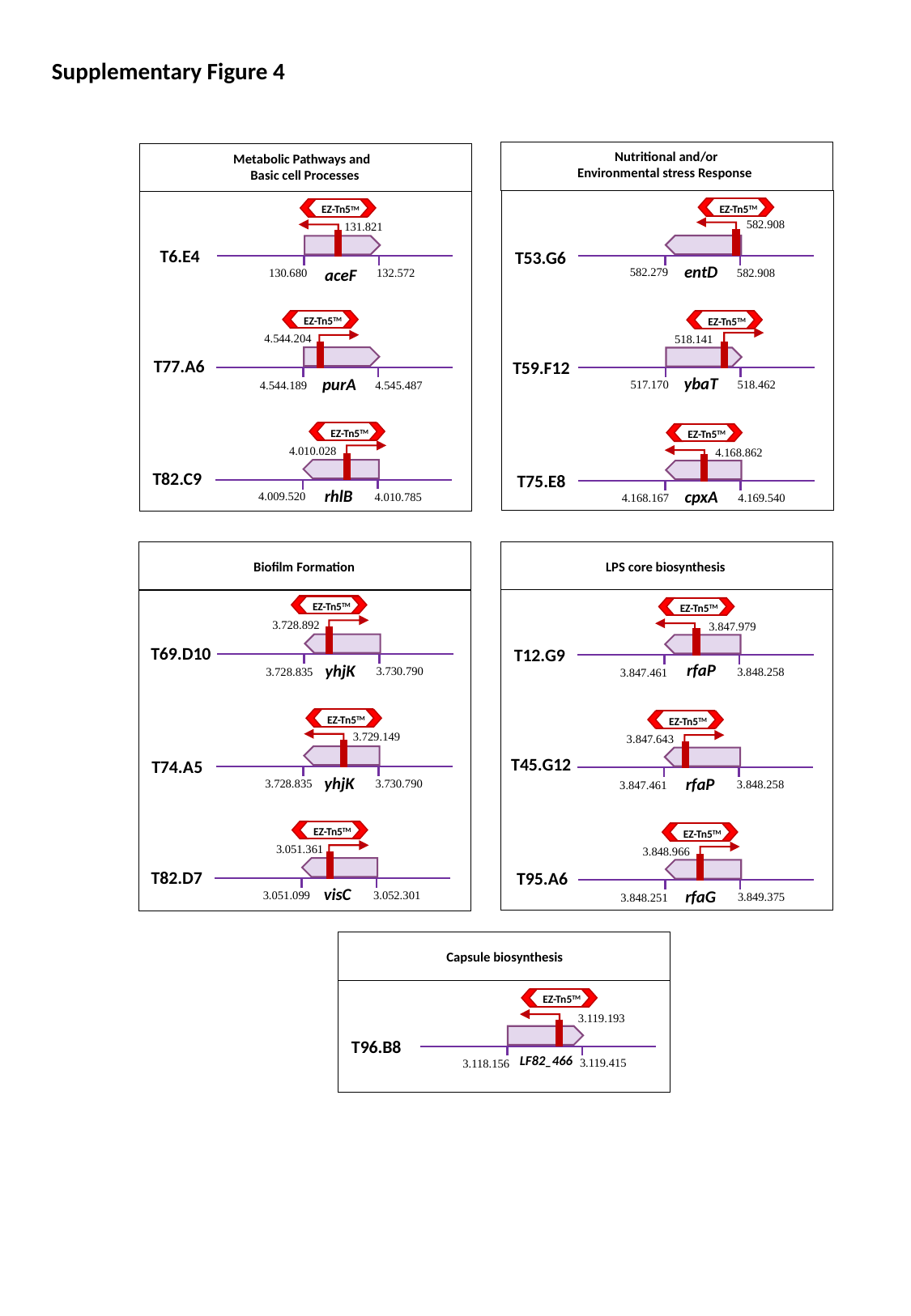

Supplementary Figure 4
Nutritional and/or
Environmental stress Response
Metabolic Pathways and
Basic cell Processes
EZ-Tn5TM
131.821
aceF
130.680
132.572
T6.E4
EZ-Tn5TM
582.908
582.279
582.908
entD
T53.G6
EZ-Tn5TM
4.544.204
4.544.189
4.545.487
purA
T77.A6
EZ-Tn5TM
518.141
517.170
518.462
ybaT
T59.F12
EZ-Tn5TM
4.010.028
4.009.520
4.010.785
rhlB
T82.C9
EZ-Tn5TM
4.168.862
4.168.167
4.169.540
cpxA
T75.E8
Biofilm Formation
LPS core biosynthesis
EZ-Tn5TM
3.728.892
3.730.790
3.728.835
yhjK
T69.D10
EZ-Tn5TM
3.847.979
3.848.258
3.847.461
rfaP
T12.G9
EZ-Tn5TM
3.729.149
3.730.790
3.728.835
yhjK
T74.A5
EZ-Tn5TM
3.847.643
rfaP
3.848.258
3.847.461
T45.G12
EZ-Tn5TM
3.051.361
3.052.301
3.051.099
visC
T82.D7
EZ-Tn5TM
3.848.966
3.849.375
3.848.251
rfaG
T95.A6
Capsule biosynthesis
EZ-Tn5TM
3.119.193
3.119.415
3.118.156
LF82_466
T96.B8

## Slide 7
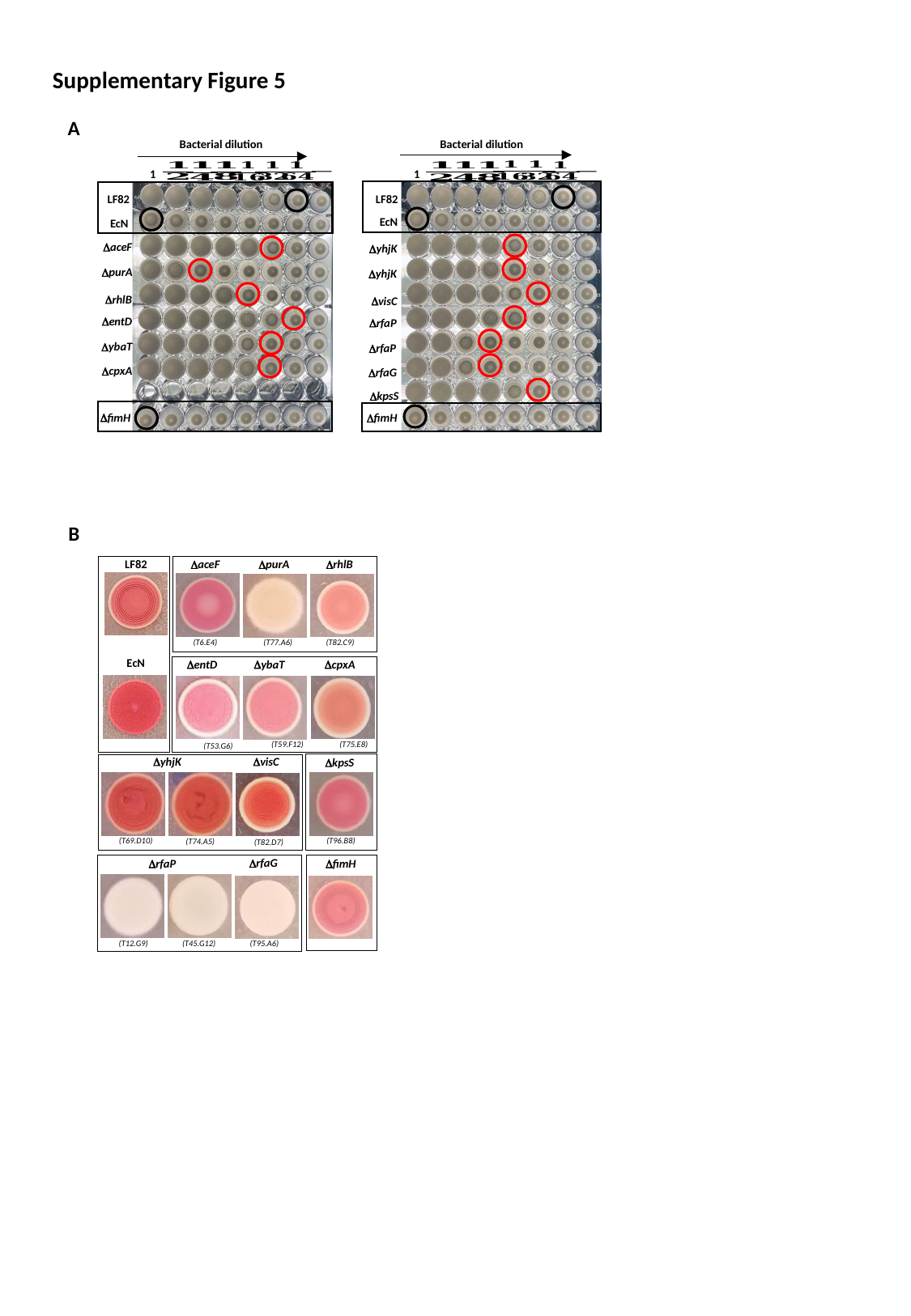

Supplementary Figure 5
A
Bacterial dilution
1
LF82
EcN
DaceF
DpurA
DrhlB
DentD
DybaT
DcpxA
DfimH
Bacterial dilution
1
LF82
EcN
DyhjK
DyhjK
DvisC
DrfaP
DrfaP
DrfaG
DkpsS
DfimH
B
LF82
EcN
DaceF
DpurA
DrhlB
(T6.E4)
(T77.A6)
(T82.C9)
DentD
DybaT
DcpxA
(T75.E8)
(T59.F12)
(T53.G6)
DyhjK
DvisC
(T69.D10)
(T74.A5)
(T82.D7)
DkpsS
(T96.B8)
DrfaG
DfimH
DrfaP
(T12.G9)
(T95.A6)
(T45.G12)

## Slide 8
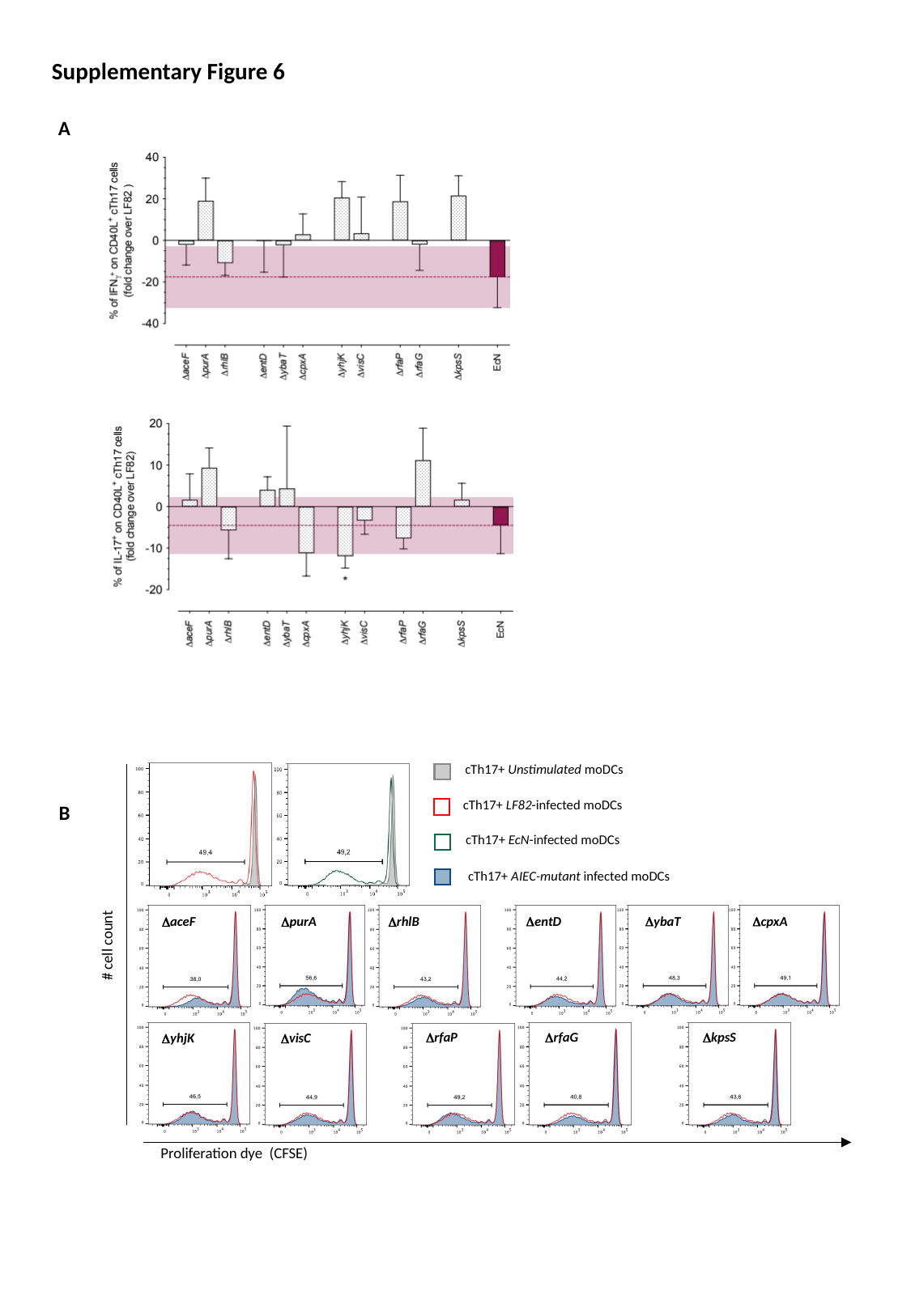

Supplementary Figure 6
A
cTh17+ Unstimulated moDCs
# cell count
Proliferation dye (CFSE)
cTh17+ LF82-infected moDCs
cTh17+ EcN-infected moDCs
cTh17+ AIEC-mutant infected moDCs
DentD
DybaT
DcpxA
DaceF
DpurA
DrhlB
DrfaP
DrfaG
DkpsS
DyhjK
DvisC
B

## Slide 9
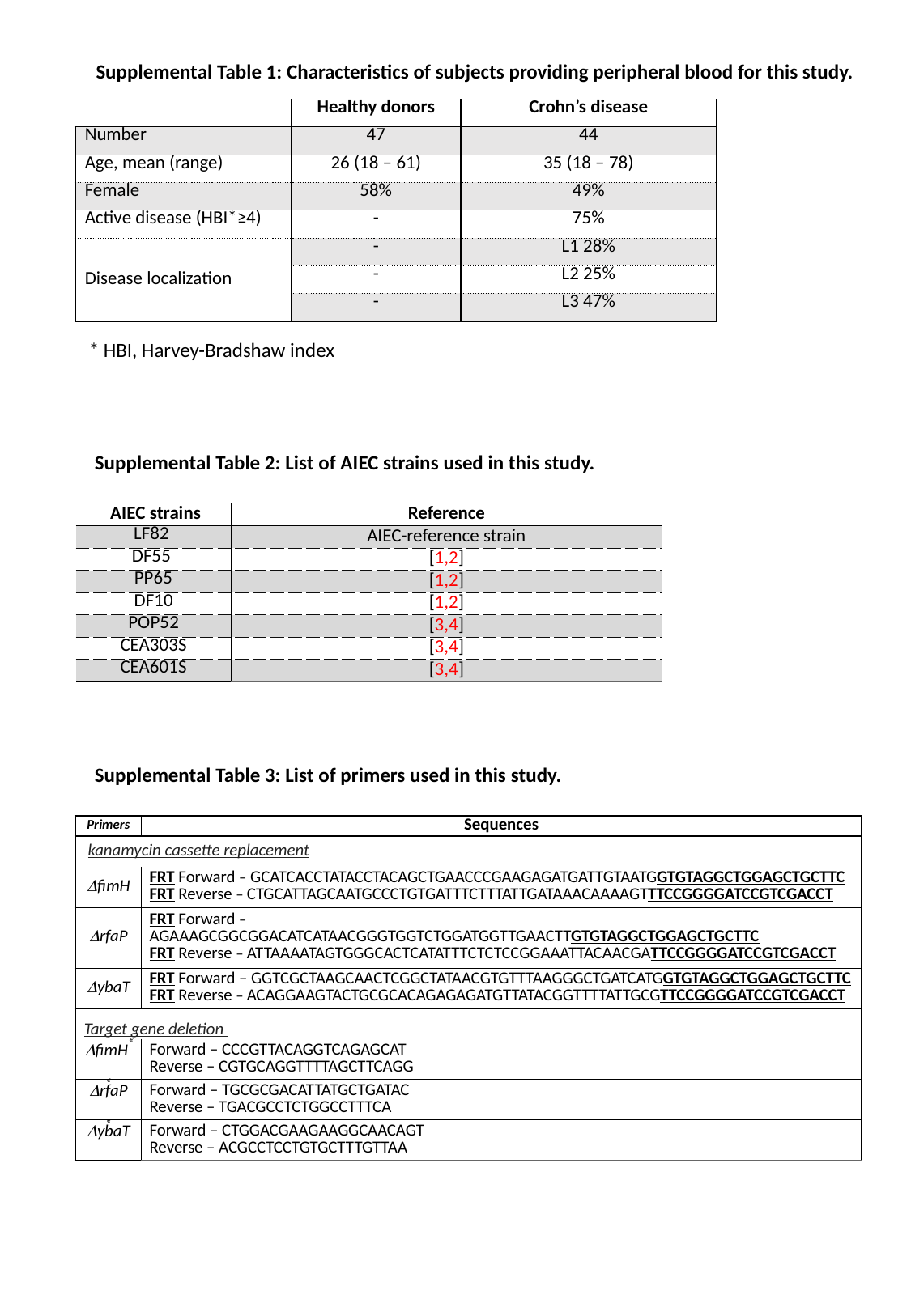

Supplemental Table 1: Characteristics of subjects providing peripheral blood for this study.
| | Healthy donors | Crohn’s disease |
| --- | --- | --- |
| Number | 47 | 44 |
| Age, mean (range) | 26 (18 – 61) | 35 (18 – 78) |
| Female | 58% | 49% |
| Active disease (HBI\*≥4) | - | 75% |
| Disease localization | - | L1 28% |
| | - | L2 25% |
| | - | L3 47% |
* HBI, Harvey-Bradshaw index
Supplemental Table 2: List of AIEC strains used in this study.
| AIEC strains | Reference |
| --- | --- |
| LF82 | AIEC-reference strain |
| DF55 | [1,2] |
| PP65 | [1,2] |
| DF10 | [1,2] |
| POP52 | [3,4] |
| CEA303S | [3,4] |
| CEA601S | [3,4] |
Supplemental Table 3: List of primers used in this study.
| Primers | Sequences |
| --- | --- |
| kanamycin cassette replacement | |
| DfimH | FRT Forward – GCATCACCTATACCTACAGCTGAACCCGAAGAGATGATTGTAATGGTGTAGGCTGGAGCTGCTTC FRT Reverse – CTGCATTAGCAATGCCCTGTGATTTCTTTATTGATAAACAAAAGTTTCCGGGGATCCGTCGACCT |
| DrfaP | FRT Forward – AGAAAGCGGCGGACATCATAACGGGTGGTCTGGATGGTTGAACTTGTGTAGGCTGGAGCTGCTTC FRT Reverse – ATTAAAATAGTGGGCACTCATATTTCTCTCCGGAAATTACAACGATTCCGGGGATCCGTCGACCT |
| DybaT | FRT Forward – GGTCGCTAAGCAACTCGGCTATAACGTGTTTAAGGGCTGATCATGGTGTAGGCTGGAGCTGCTTC FRT Reverse – ACAGGAAGTACTGCGCACAGAGAGATGTTATACGGTTTTATTGCGTTCCGGGGATCCGTCGACCT |
| Target gene deletion | |
| DfimH | Forward – CCCGTTACAGGTCAGAGCAT Reverse – CGTGCAGGTTTTAGCTTCAGG |
| DrfaP | Forward – TGCGCGACATTATGCTGATAC Reverse – TGACGCCTCTGGCCTTTCA |
| DybaT | Forward – CTGGACGAAGAAGGCAACAGT Reverse – ACGCCTCCTGTGCTTTGTTAA |
